# Microwave-Assisted Synthesis: Can Transition Metal Complexes Take Advantage of This “Green” Method?

**DOI:** 10.3390/molecules27134249

**Published:** 2022-06-30

**Authors:** Elisabetta Gabano, Mauro Ravera

**Affiliations:** 1 Dipartimento per lo Sviluppo Sostenibile e la Transizione Ecologica, Università del Piemonte Orientale, Piazza Sant′Eusebio 5, 13100 Vercelli, Italy; elisabetta.gabano@uniupo.it; 2 Dipartimento di Scienze e Innovazione Tecnologica, Università del Piemonte Orientale, Viale T. Michel 11, 15121 Alessandria, Italy

**Keywords:** microwave heating, metal complexes, organometallic compounds, synthesis

## Abstract

Microwave-assisted synthesis is considered environmental-friendly and, therefore, in agreement with the principles of green chemistry. This form of energy has been employed extensively and successfully in organic synthesis also in the case of metal-catalyzed synthetic procedures. However, it has been less widely exploited in the synthesis of metal complexes. As microwave irradiation has been proving its utility as both a time-saving procedure and an alternative way to carry on tricky transformations, its use can help inorganic chemists, too. This review focuses on the use of microwave irradiation in the preparation of transition metal complexes and organometallic compounds and also includes new, unpublished results. The syntheses of the compounds are described following the group of the periodic table to which the contained metal belongs. A general overview of the results from over 150 papers points out that microwaves can be a useful synthetic tool for inorganic chemists, reducing dramatically the reaction times with respect to traditional heating. This is often accompanied by a more limited risk of decomposition of reagents or products by an increase in yield, purity, and (sometimes) selectivity. In any case, thermal control is operative, whereas nonthermal or specific microwave effects seem to be absent.

## 1. Introduction

Since the first reports in the second half of the 1980s [1], the use of microwave (MW) heating has been growing significantly over the years with many applications in the laboratory (e.g., solid-state chemistry, nanomaterial synthesis, and, above all, organic synthesis and drug discovery).

Microwave-assisted synthesis (MAS) is generally characterized by higher yields, higher selectivity, milder reaction conditions, and shorter reaction times compared to conventional heating (CH) [2,3]. In addition, it is considered an effective approach to green and sustainable chemistry due to its environmentally friendly features [4]. The use of MWs as an alternative energy source allows less time-consuming synthesis because of rapid heating and transfer of energy to the reaction medium, permits the employment of eco-friendly solvents or solvent-free conditions, and favors catalytic transformations [5,6]. MAS can fit at least two of the “Twelve Principles of Green Chemistry” introduced in 1998 by Paul Anastas and John Warner (i.e., “Safer solvents and auxiliaries” and “Design for energy efficiency”) [7].

For all these advantages and its potential, MW heating was called “the Bunsen burner of the 21st century” [8]. However, if, on the one hand, MWs have become an essential tool in all areas of synthetic organic chemistry, on the other hand, it seems that in coordination chemistry, they do not have the same importance.

## 2. Microwave and Chemistry: Background Information

MWs fall between radio and infrared frequencies (from around 0.3 to 300 GHz), so it cannot be said that it is one of the most energetic ranges [6,9,10]. Although already known as a source of energy, MW irradiation was used rarely to assist in laboratory syntheses up to the 1980s and early 1990s. Little by little, the advantages of MAS over the CH have become increasingly evident.

**Figure 1:**
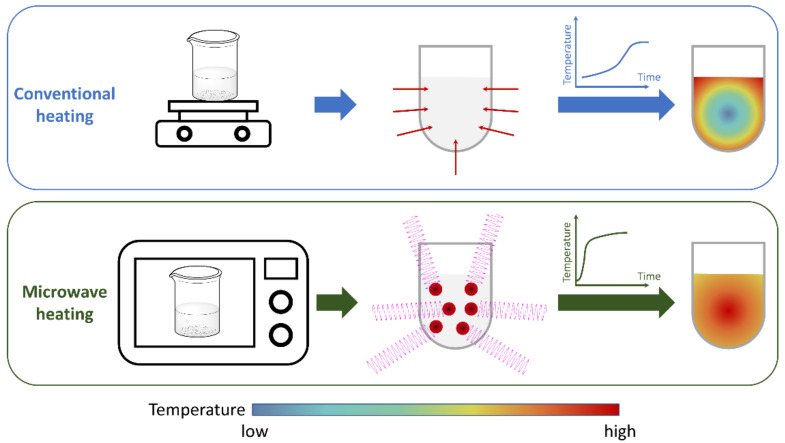
Schematic difference between conventional and microwave heating.

In CH, heat is transmitted via conduction, convection, or radiation. Hotplates and heating mantles transfer thermal energy into a reaction mixture by warming the mantle or plate, which in turn heats the vessel, and the vessel then warms the reaction mixture, often with thermal gradients. The mechanism of energy transfer in a MAS is different because energy is transferred directly and instantaneously to the components of the reaction mixture independently of their position within the vessel (Figure 1) [11].

The dielectric properties of a substance affect its interaction with the electric field of the MW since it will interact with polar or ionic molecules. Oscillations of the field cause molecules to rotate aligning with the field itself, according to the “dipole rotation” mechanism for polar molecules or the “ionic conduction” mechanism for ionic species. As the molecules move, they generate heat, leading to the rapid temperature rise.

The dielectric constant (ε′) measures the ability of a molecule to store electromagnetic energy through polarization. Molecules with large dipole moments also have large dielectric constants because the polarization depends on the dipole rotation when aligning with electric field. The dielectric loss (ε″) is related to the ability to convert energy into heat (i.e., it represents the amount of MW energy that is lost as heat). The ability of a substance to convert electromagnetic energy into heat at a given frequency and temperature is determined by the loss tangent, tan δ = ε″/ε′ (i.e., the dissipation factor of the sample, which is a measure of the conversion of MW into thermal energy). In simple terms, the more polar a substance is, the greater its ability to couple with the MW energy is, leading to a rapid rise in temperature [12,13].

Each solvent and reagent can absorb MW energy differently depending on their polarity, so the absorbance of the whole reaction mixture is related to all its components. Therefore, it is clear that when syntheses are performed in solution, the choice of the solvent plays a crucial role. High-absorbing solvents have ε″ greater than 14 and heat up very quickly within the MW reactor; examples of this kind of solvents are dimethyl sulfoxide (DMSO), nitrobenzene, and small-chain alcohols, such as methanol (MeOH) and ethanol (EtOH). Medium absorbers have ε″ between 1 and 14 and heat up very efficiently in longer time; dimethylformamide (DMF), acetonitrile, butanols, ketones, and water belong to this category. Finally, low-absorbing molecules have ε″ that are less than 1 and do not undergo significant heating unless it occurs in a much longer time; this family of solvents is represented by chloroform, dichloromethane, ethyl acetate, tetrahydrofuran (THF), and, as expected, ethers and hydrocarbons [13].

The polarity of the solvent is not the only factor in determining the absorbance of microwave energy, but it is a useful guideline. Usually, when a high temperature is needed, a very polar solvent is used to heat the mixture very rapidly. On the contrary, when MW-transparent solvents are employed, often, other substances in the reaction mixture will contribute to the overall temperature. Such molecules, which act as “molecular radiators” for MW radiation, may also have enhanced reactivity [2]. Moreover, solvents, which do not couple very well to MW radiation, can function as a heat sink. Therefore, temperature-sensitive reaction mixtures can take advantage of this since internal temperature remains low [13].

Ionic liquids, which are compounds entirely composed of ions with a melting point below 100 °C, are promising substitutes for common organic solvents [14]. Ionic liquids absorb MWs efficiently and rapidly transfer energy by ionic conduction [13].

Nowadays, the common belief is that the observed rate enhancements are merely a thermal/kinetic effect because of the temperatures that can be quickly obtained in the vessels when the reaction mixture is irradiated in an MW field. Furthermore, overheating of polar liquids of 13–26 °C above the usual boiling point can occur due to the “superheating” effect [2].

However, in particular in the early days of MAS, the experimental results could not always be explained by rapid heating alone. Therefore, the nonthermal effects of MW have been suggested even though this point is rather controversial and difficult to be demonstrated. Kappe et al. in 2013 concluded that “the existence of so-called nonthermal or specific microwave effects is highly doubtful” and that “microwave chemistry is not ‘voodoo science’” [15]. In any case, possible nonthermal effects resulting from interaction between MW and molecules, if any, are difficult to distinguish as a single contribution to the final result [2,16,17].

According to the standards of the International Telecommunication Union, the frequency 2.45 GHz is available for domestic and commercial MW ovens. This frequency is also the most popular because of the existence of a relatively inexpensive and compact MW oscillator tube, the magnetron, which contributed to the market expansion of the 2.45 GHz band [18].

Initially, the instruments used in the laboratory were commercial domestic ovens, which are multimode reactors with large cavities in which the MW field is distributed in a chaotic manner. Such ovens lack stirring and control of the temperature and of the amount of power applied, thus resulting in low reproducibility, spilling of the vessel content, or even explosions. In many (old) papers, the modification of such commercial apparatuses to make them more suitable for chemical syntheses is often reported [19].

In order to address the need of testing more easily single reactions on the milligram scale, single-mode MW systems were designed in the late 1990s. The main difference between the new single-mode and the previously existing multimode design is the generation of a single-mode of energy during the irradiation cycle. The MW generates pockets of high energy and low energy as the moving wave either reinforces or cancels. This leads to the presence of high-energy fields, low-energy fields, and a point where the amount of energy is equal to zero, called the node [20].

The single-mode cavity is designed for the length of only one wave, therefore generating only one mode of MW energy. The wave generates a center of high electromagnetic field intensity with a homogenous energy distribution in the cavity where the synthesis takes place. In a multi-mode system, there are many centers of high electromagnetic intensity, called “hot spots”, but there are also several low-energy spots, creating “cold spots”.

The presence of different hot spots results in a higher temperature in some points of the mixture rather than in the bulk system. Hot spots may also arise from differences in dielectric properties of substances in the reaction mixture [2].

As already mentioned, the use of MW irradiation has become increasingly widespread and is today a mature technology also considering the improvement obtained in flow and scale-up chemistry [10,13,21,22,23,24,25,26,27,28,29,30,31,32,33,34]. Complete coverage of the applications in the field of organic chemistry is beyond the scope of this review. However, it is worth mentioning the synthesis of heterocycles because of their importance in pharmaceutical chemistry, polymer synthesis, and material science. Another field of application of MWs are the multicomponent organic syntheses due to their potential to provide an efficient and faster way to increase the molecular complexity and diversity necessary in high-throughput chemistry (e.g., combinatorial chemistry, parallel synthesis) [35], not to mention the modification of the chemo-, regio-, and stereo-selectivity of an MW-assisted reaction in relation to CH [36].

MW irradiation has been widely used in the case of metal-catalyzed synthetic procedures [13,37,38,39,40,41,42,43,44,45,46,47,48]. This includes also facile, green, and useful click reactions, which are characterized by the formation of a single product in high yield, the elimination of by-products, atom economy, the use of mild reaction conditions, water compatibility, and the use of simple purification processes [49,50,51,52].

However, the real advantages of MW irradiation can be easily observed in biomedical applications. Current technology allows temperatures compatible with heat-sensitive biological molecules, such as in the case of reactions involving carbohydrates, nucleosides, peptides, proteins, and peptoids, but also polymerase chain reaction, trypsin digestion, and solid-phase peptide synthesis [24,28,53]. MWs allow an efficient energy transfer to the molecules instead of a method of rapidly heating them to high temperatures, decreasing the risk of loss of activity or degradation (“Think of a microwave as a scalpel compared with a sledgehammer” [54]).

Finally, MWs offer some distinctive advantages in material synthesis. The possibility of selective and homogenous heating of the reactants in MAS minimizes thermal gradients and provides uniform nucleation and growth conditions that lead to the formation of more uniform nano/materials in terms of size distribution, nucleation, crystal growth processes, and so on [55,56,57,58,59,60].

In contrast, despite the strong impulse given to the field by the pioneering work of Mingos and coworkers [61,62,63,64,65,66], MW heating was less successful in the “simple” synthesis of metal compounds [67].

In the next sections, a critical analysis of the literature data will be presented with the aim of evaluating the impact of MW irradiation on the synthetic chemistry of transition metal complexes and organometallic compounds, also including new, unpublished results. Metals will be divided according to their group in the periodic table. As far as many papers report the synthesis of compounds containing different metals, the description will appear for the first metal encountered and will be recalled briefly for the others later.

## 3. Early Transition Elements of Groups 5–7

### 3.1. Vanadium

**Figure 2:**
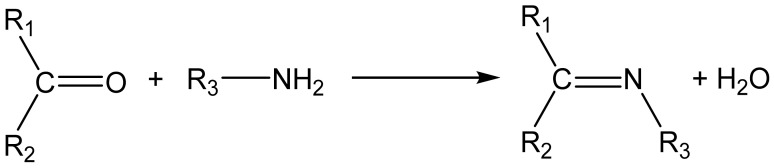
General scheme for the formation of Schiff bases.

Schiff bases are versatile organic compounds known since the mid-19th century; their coordination chemistry attracted a great deal of attention because of their significance in organic synthesis, analytical chemistry and also in the refining of metals, electroplating and other fields. Traditionally, Schiff bases are simply prepared by refluxing mixtures of an amine and a carbonyl compound (aldehyde or ketone) in an organic solvent (Figure 2).

Similar procedures can be used to obtain the metal complexes by refluxing preformed Schiff bases or their components and metal salts. For this reason, the simple, cost-effective, and versatile route represented by MWs was attempted to obtain cleaner reactions in a shorter time and, hopefully, better yields.

**Figure 3:**
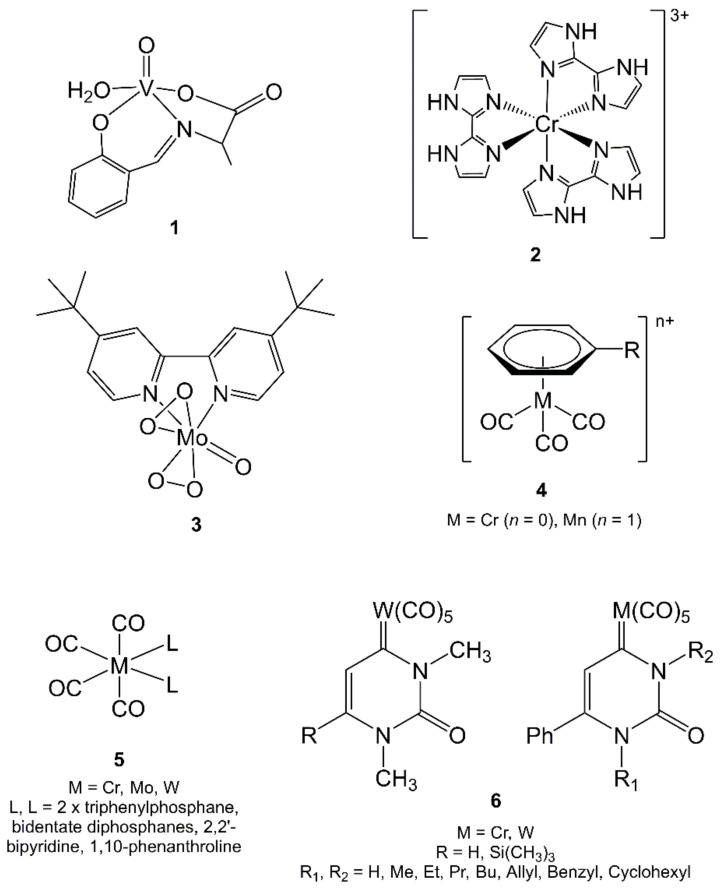
A selection of complexes containing V, Cr, Mo, W, and Mn cited in the text.

The template synthesis of complexes with Schiff base was performed by reacting salicylaldehyde or *o*-hydroxyacetophenone, an amino acid (i.e., glycine, alanine, lysine, arginine, and phenylalanine), and VOSO_4_ at pH between 5.5–5.8 in water/EtOH by using MW (210–240 W, 2–3 min at steps of 10 s to reach a temperature of 70 °C) (see example **1**, Figure 3). With this procedure, good-quality crystals were obtained directly “in summer days in the Indian tropical climatic conditions” (the gradual and slow natural cooling favored the crystallization; yields ranged from 56 to 87%) [68].

Another Schiff base derived from *o*-vanillin with 6-(trifluoromethoxy)benzothiazole and its metal complexes with VO(II) and ZrO(II) (1:2 M:L ratio) but also Cr(III), Mn(II), Fe(II), Co(II), Ni(II), Cu(II), Zn(II), Cd(II), and Hg(II) (1:1 M:L ratio) were synthesized using MW radiation (110 W for 1 min). In the conclusion, the authors claimed that “the microwave-assisted syntheses have been found to be much easier, convenient, quicker and eco-friendly”. However, no comparison with other synthetic methods was reported, and the declaration seems to be only a general statement [69].

### 3.2. Chromium, Molybdenum and Tungsten

In the realm of Schiff bases, two other papers reported the use of MWs in the synthesis of both the ligands and their complexes, but in these cases, a comparison with traditional heating was present. Reactions of 5-bromosalicylaldehyde with 2-amino-5-nitrothiazole and 4-dimethylaminobenzaldehyde with 2-amino-3-hydroxypyridine were performed by both CH and MAS. The two methods were also applied to the formation of the Cr(III), Co(II), Ni(II), and Cu(II) complexes of the ligands obtained from the previous reaction. The ethanolic mixtures of the organic reagents, refluxed for 3–4 h, gave the desired ligands in 70–72% yield, whereas the reagents irradiated for 4–5 min in an MW oven gave 87–88% of the same ligands. A similar behavior was observed in the synthesis of the complexes. Ethanolic mixtures of metal ions and Schiff bases in a 1:2 (M:L) ratio refluxed for 6–10 h yielded 60–70% of the complexes, whereas the MW irradiation was completed in a shorter time (7–10 min) to give a yield of 77–84% [70].

Similar conditions and results were used in the reaction between 5-bromosalicylaldehyde and 4-nitro-1,2-phenylenediamine (thermal reaction in refluxing MeOH, 6 h, 73% yield vs. MW irradiation in EtOH, 5–6 min, 91% yield) and the 1:1 complexes obtained from the resulting Schiff base with Cr(III), Co(II), Ni(II), and Cu(II) (thermal reaction in refluxing MeOH, 7–10 h, 62–68% yield vs. MW irradiation in EtOH, 6–9 min, 79–85% yield) [71].

Four heterocyclic ketimines were prepared by the condensation of 2-acetylfuran and 2-acetylthiophene with thiosemicarbazide and semicarbazide hydrochloride in MeOH by using MWs and CH. Subsequently, Cr(III) complexes were prepared by mixing CrCl_3_ in 1:1 and 1:2 mole ratios with monobasic bidentate ketimines under both conditions. Thermal reactions were completed in hours (3.5–4 h for the ligands, 12–15 h for the complexes in refluxing MeOH), whereas MWs produced the final products in minutes (4–7 min for all compounds; scarce information on the experimental setup was provided). An increase in terms of yield was observed in favor of MWs (ranging from +6 to +19%), with a concomitant decrease of the amount of solvent used for the synthesis (from 30–100 mL under CH to 2–5 mL under MWs) [72].

Tris(*N*,*N*-diimine)chromium(III) complexes are known for their potential as photosensitizers in photodynamic antimicrobial chemotherapy (PACT), a relatively new method that utilizes the combined action of light, oxygen, and a photosensitizer to bring about the destruction of bacterial and fungal infections. The synthesis of such complexes is usually a very time-consuming procedure. The MW assisted synthesis of [Cr(2,2′-bimidazole)_3_](NO_3_)_3_ (**2**, Figure 3) was carried out by irradiating a mixture of CrCl_3_ and [Ag(2,2′-biimidazole)](NO_3_) in 10 mL of THF for 90 s at 110 °C and 300 W power (yield = 94%) [73].

Miscellaneous Cr(III), Ru(III), Ir(III), Pt(II), and Au(III) complexes were synthesized in a few minutes (instead of hours or days) in moderate to good yields in an MW oven (500–650 W) by using a Teflon autoclave [62].

The MAS was used to obtain other sparse Mo coordination compounds. The oxodiperoxo complex [MoO(O_2_)_2_(tbbpy)] (tbbpy = 4,4′-di-*tert*-butyl-2,2′-bipyridine) (**3**, Figure 3) was isolated from the reaction of [MoO_2_Cl_2_(tbbpy)] in water under MW-assisted heating at 120 °C for 4 h with a yield of 12%, using only air as the oxygen source. Importantly, when the same reaction was carried out under conventional reflux, no oxodiperoxo complex was formed [74].

Bis[tris(2-ammonioethyl)amine] bis(pentafluoridooxidomolybdate) difluoride monohydrate, (C_6_H_21_N_4_)_2_[MoOF_5_]_2_F_2_·H_2_O), was prepared by reacting MoO_2_, tris(2-aminoethyl)amine, HF, and EtOH using Teflon autoclaves installed in an MW oven at 190 °C for 1 h. Because the paper focused on the X-ray structure, no further information about the MAS was provided [75].

The chemistry of metal carbonyls has attracted considerable interest for several decades not only because of their basic aspects, including the reactivity toward several classes of organic ligands, but also for their applications in catalysis or as a source of zerovalent metals. A general difficulty in performing transition metal–carbonyl chemistry is the relative inertness of the metal–carbonyl bond, which often makes reaction times annoyingly long [76]. For example, the study of the chemistry of (η^6^-arene)chromium carbonyls has been historically limited by the high temperature and long reaction times required for their synthesis, which, in turn, decreases the yields. The reaction between Cr(CO)_6_ and various arenes in THF under MW irradiation (300 W, 1.5 h at 160 °C) provided a reasonable to high yield of the (η^6^-arene)tricarbonylchromium compounds (**4**, Figure 3) (48–79% depending on the arene). These yields were sometimes comparable to those obtained in conventional prolonged thermal reactions [77].

An (almost) conventional MW oven (750 W) was used to synthesize twenty group 6 organometallic compounds in diglyme, starting from [M(CO)_6_] (M = Cr, Mo, W) in a 100 mL round-bottomed flask connected to a water condenser. The reactions generally proceeded without an inert atmosphere, in high yields, and with short reaction times. For example, *cis*-[Mo(CO)_4_(dppe)] [dppe = ethane-1,2-diylbis(diphenylphosphane)] was prepared in >95% yield in 20 min. Similarly, the reaction of Mo(CO)_6_ with dicyclopentadiene afforded [Mo(η^5^-C_5_H_5_)(CO)_3_]_2_ (C_5_H_5_^−^ = cyclopentadienido, also indicated as Cp) in a simple one-step synthesis with >90% yield, whereas reactions with Cr(CO)_6_ generally required an inert atmosphere and proceed less cleanly [78].

A modified Chatt procedure, using NaBH_4_ as catalyst, was employed to synthesize several group 6 tetracarbonyl phosphane and tertiary amine complexes [M(CO)_4_L_2_] (M = Cr, Mo, W, L_2_ = 2 × triphenylphosphane, bidentate diphosphanes, 2,2′-bipyridine, 1,10-phenanthroline, **5**, Figure 3) by MW heating (400 W) in various alcohols as solvents. The combination of alcohols and borohydride salts provided an ideal set of reaction conditions for the application of MW heating. The alcohol hydroxyl group strongly absorbs the microwaves via the dipolar absorption mechanism, and the borohydride salts absorb through the ion conduction mechanism, resulting in a rapid temperature increase of the reaction mixture. In fact, heating times were greatly reduced from 300 to 3–40 min, whereas yields did not improve significantly. Interestingly, the mild, rapid reaction conditions allowed one to selectively isolate the *cis*-[Mo(CO)_4_(triphenylphosphane)_2_] complex directly from [Mo(CO)_6_] [79].

The molybdenum and tungsten tetracarbonyl complexes containing the ligand ethyl [3-(2-pyridyl)-1-pyrazolyl]acetate were prepared rapidly and in one step from the [M(CO)_6_] starting materials with MW heating in a diglyme-toluene mixture by using a closed 100 mL Teflon vessel. The yields were comparable with those achievable by the traditional preparation routes (thermal: 3 h in toluene at 50 °C in two steps, 80% yield; MW: 300 W at 180 °C for 30 s, 63% yield). A longer reaction time was required for the formation of the tungsten complex due to the lower reactivity of [W(CO)_6_] (85% yield at 180 °C for 15 min, 600 W). In addition to shorter reaction times, MW syntheses required relatively small quantities of solvents, and it was not necessary to use an inert atmosphere [80].

Mono and disubstituted ureas reacted with the alkynyl Fischer carbene complexes of Cr and W to give mono- and di-*N*,*N*-substituted organometallic uracil analogues (**6**, Figure 3), under CH (60 °C in THF) and MW heating. In general, thermal reactions required reaction times >30 min depending on the reagents, whereas MWs (400 W) required 30 min or less. The yields under MW irradiation were similar with respect to the thermal ones [81].

A particular subfield of (potential) applications of the MW heating includes the reactions of metals in liquid media. In this case, arcing represents a severe problem (a well-known phenomenon faced by those who have introduced by mistake a metallic object into a domestic MW oven). It has been demonstrated that the use of low MW power and polar solvents with high viscosity and high boiling points as well as an efficient stirring of a very fine metal powder reduces the amount of arcing. Several reactions were chosen from the literature to give a representative range of reactions involving metal powders; in particular, Cr was reacted in an open vessel with refluxing toluene or benzene by using a modified MW commercial oven to give the [Cr(η^6^-arene)_2_][BPh_4_] complexes. The results showed that the use of MW heating does not offer any appreciable advantage over CH in terms of reaction yields. However, the refluxing conditions are reached relatively quickly with respect to the use of heating mantles or oil baths, reducing the overall reaction time by as much as 25% when compared to CH [82].

In a time when commercial apparatus and glassware for MW applications were rather uncommon, MASs of well-known complexes were used to test new equipment. Baghurst and Mingos proposed a thick-walled Pyrex reaction vessel that resembles the Fischer–Porter pressurizable glass reactor. The new reaction vessel was intended to be inserted into the MW oven using a suitably designed port and the more durable glass can bypass the limitations of Teflon vessels (i.e., the use of high-boiling solvents, longer reaction times, etc.). To evaluate the new vessel, [Mo_2_(acac)_4_] (acac = acetylacetonato) and [Mo_6_Cl_8_][CH_3_COO]_2_Cl_2_ as well as one Rh and two Ru complexes were used. The new equipment overcame many of the disadvantages associated with the Teflon vessels. The possibility of reaching high pressures resulted in a superheating of the reaction by approximately 40–60 °C, and the reaction times decreased by a factor of about 100, with a concomitant increase in yield [65].

More recently, a scientific monomodal MW apparatus was interfaced with a commercially available Raman module for the in situ, real-time monitoring of organometallic reactions. A fiber optic probe attached to the Raman module was introduced into the MW cavity, the laser was focused via a quartz light tube positioned a few mm from the reaction vessel, and the monitoring of the ligand substitution reactions of [Mo(CO)_6_] was used as a proof-of-concept [83]. Nowadays, commercial scientific MW ovens have evolved so much that they only share the basic principles with the household or lab-modified equipment used in the prehistory of the method. During their development, MW instruments incorporated some of the tricks that were suggested by the pioneers of the technique [84].

### 3.3. Manganese

Four ligands (i.e., one substituted ethane-1,2-diamine and three benzene-1,2-diamines) were reacted with Mn(II) in 1:1 mole ratio by using an MW oven for 2–6 min at 600 W. The different “ML” complexes were obtained with 25–65% yield (CH gave the same complexes in 20–40% but with reaction times of 2–3 h) [85].

The synthesis of metal complexes containing Schiff bases as ligands is often characterized by a systematic use of transition metal ions from different groups. Mn(II) ions as well as Cu(II), Ni(II), Co(II), Zn(II), Hg(II), and Sn(II) were reacted with 1-(2-furyl)-3-(4-aminophenyl)-2-propene-1-one, exploring both CH and MW synthesis (metal acetates and ligand in refluxing EtOH for 5 h vs. MW at 600 W for 1–2 min). The ligand and complexes were produced by MAS in higher yields (the yields of the CH were between 75–85%, whereas MAS gave 90–95% values) [86].

In another experiment, the metal complexes were obtained by reacting together (one pot) the three components of the Schiff base, which are the aldehyde (i.e., 2-hydroxy-3-methoxybenzaldehyde), the amine (i.e., methylamine or ammonia), and the transition metal salts (i.e., Mn(II) and Zn(II)) in water. Complexes [Mn_7_(mimmp)_6_(OH)_6_][ClO_4_]_2_ and [Zn_7_(mimmp)_6_(OH)_6_](NO_3_)_2_ (mimmp = 2-methoxy-6-methyliminomethylphenol) were obtained after MAS (in a 60 mL Teflon-lined autoclave, 80 °C, 300 W, and pressure = 6–7 atm for a total of 5 min) with yields of 27% and 20% for Mn and Zn, respectively (CH conditions: 15 mL Teflon-lined autoclave, 80 °C, for 120 h, yields = 21 and 15%) [87].

A further example of the MAS of a Schiff base complex with Mn(II), together with other metal ions, is reported in Section 3.1 [69].

High nuclearity transition metal complexes have attracted great interest due to their relevant magnetic properties and applications in fields such as information storage, quantum information processing, or magnetic cooling. Synthetic methods used to obtain cluster complexes are usually straightforward and based on the self-assembly of low-nuclearity compounds under controlled experimental conditions. Therefore, it was natural to extend the MAS to obtain these kinds of products. For example, a mixture of Mn(ClO_4_)_2_, salicylaldoxime and sodium methoxide in MeOH was reacted in an MW reactor in a sealed glass tube (110 °C, power = 200 W, pressure about 7.5 atm, for a total of 5 min). After cooling (1 min), green-black crystals of the all-Mn(III) single-molecule magnet [Mn_6_(CH_3_OH)_4_O_2_(O_2_CH)_2_(salicylaldoxime)_6_]·2MeOH started to form immediately, and after 24 h, the yield was ≈80%. The same complex could also be made without MW irradiation under ambient conditions, but crystalline material did not appear immediately, and the maximum yield of ≈30% was only achieved after a 60 min reaction and a 5 d crystallization period [88].

The reaction of MnCl_2_, NiCl_2_, 3,5-di-*tert*-butylsalicylic acid, and 3-dimethylamino-1-propanol was studied in an acetonitrile/MeOH mixture and in the presence of a weak base under MW irradiation (250 W MW pulse for 5 min at 140 °C). When triethylamine was used, a small metal cluster containing a [Mn_7_] core was obtained after crystallization. On the contrary, in the presence of isopropylamine, a mixture of [Mn_7_] and a [Mn_2_Ni_2_]-based compound was obtained. Interestingly, the weak base used to deprotonate the carboxylic acid was not an innocent player in this reaction. Unfortunately, MAS has been a useful tool to separate mixtures or to promote the formation of one pure product [89].

Finally, as in the case of group 6 metals, MW heating was applied to the synthesis of Mn-arene carbonyl complexes. The most convenient method for the synthesis of [Mn(η^6^-arene)(CO)_3_]^+^ complexes (**4**, Figure 3) is the AlCl_3_-catalyzed exchange between [MnBr(CO)_5_] and the liquid arene as solvent or arene dissolved in decalin (at 100 °C for at least 4 h). The same synthesis was attempted in a domestic MW apparatus (850 W) by irradiating [MnBr(CO)_5_] and the arene in 1,2,4-trichlorobenzene and Al powder for 3 min. The yields were a little disappointing, being about half of those of the conventional syntheses. Similarly, sterically hindered [Fe(η^6^-arene)(η^5^-cyclopentadienido)][PF_6_] complexes with *tert*-butyl substituents were also prepared [90].

### 3.4. Technetium-99m and Rhenium

Reaction speed, as well as clean reaction mixtures to limit purification steps, is of paramount importance when radioactive isotopes are manipulated, in particular for in vivo uses. ^99m^Tc is widely employed as a radioactive tracer for nuclear medicine, and it is obtained from a ^99^Mo/^99m^Tc generator as pertechnetate (^99m^TcO_4_^−^) that needs to be reduced and complexed before administration. A typical clinical kit reaction involves the addition of ^99m^TcO_4_^−^ to a vial containing a lyophilized mixture of the ligand, a reducing agent (in general Sn(II)), and various buffers and stabilizers. As the half-life of ^99m^Tc is only 6 h, it is mandatory to obtain the maximal radiochemical purity (RCP) as soon as possible; this means that the overall reaction (reduction + complexation) must be complete and fast.

**Figure 4:**
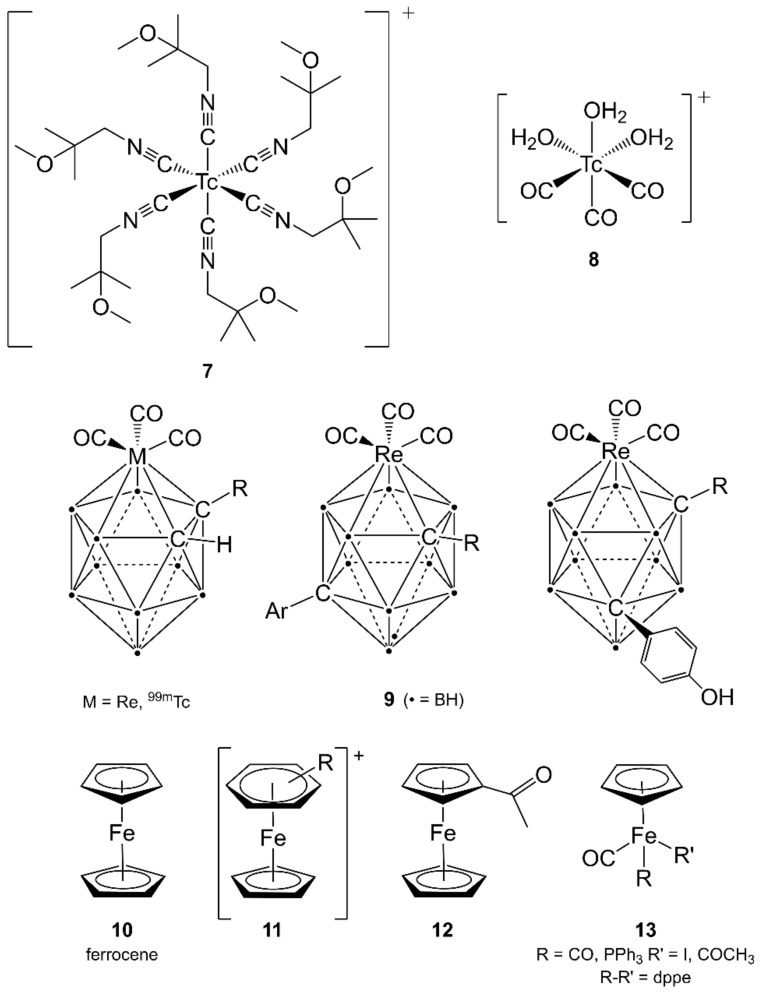
A selection of complexes containing Tc, Re, and Fe cited in the text.

^99m^Tc sestamibi (Cardiolite^®^, **7**, Figure 4) is a cationic radiotracer approved as a myocardial perfusion agent to visualize blood flow through the heart and is prepared using the water bath method by mixing ^99m^TcO_4_^−^, Sn(II), and the ligand as tetrakis(2-methylisobutylisonitrile) copper(I) tetrafluoroborate. A variety of alternative techniques have been proposed to warm the vial, with the main goal of bringing the kit to a boil for 10 min. It is clear that MW heating may represent one of these alternatives, and consequently, it was proposed to prepare ^99m^Tc-sestamibi [91]. It takes approximately 20 s of heating time in an MW oven (450 W) to make the overall reaction with an average RCP of 97% (this reaction time was later reduced to 10 s) [92,93]. The MW heating was also proposed for the synthesis of tetrakis(2-methylisobutylisonitrile)copper(I) tetrafluoroborate (in a domestic microwave oven at 240 W for 25 s in 68% yield) [94].

Another radiochemical tracer is ^99m^Tc bicisate (bicisate = *N*,*N′*-1,2-ethylene-di-yl-bis-L-cysteinate diethyl ester). It is a brain-imaging agent approved for localization of stroke in patients and detection of cerebral ischemia, seizures, and brain trauma. The commercial Bicisate kit is similar to that used to prepare ^99m^Tc-sestamibi. After mixing the ligand, reducing agent, and ^99m^TcO_4_^−^, the solution stands for 30 min at room temperature before use to obtain the highest RCP. In addition, in this case, MW irradiation has been suggested to shorten the reaction time. It was demonstrated that that the best heating temperature to obtain the ^99m^Tc-bicisate preparation was ≈70 °C and that the final radiolabeling results were the same using a hot water incubator or an MW oven at 300 W for 8 s. However, the MW oven is a better choice because of the faster and more uniform heating. With this protocol, a radiochemical purity >95% was obtained within 5 min post reconstitution [95].

The MW heating was evaluated in a passage of the multistep platform to produce molecular imaging and therapy agents based on the carbonyl precursor [M(CO)_3_(OH_2_)_3_]^+^ (M = ^99m^Tc and Re, the latter being the “cold” model for “hot” ^99m^Tc and the therapeutic isotopes ^186/188^Re). The starting material was obtained from MO_4_^−^ in 3 min in close to quantitative yield at 130 °C (^99m^Tc) or 150 °C (Re) under MW irradiation (20 min with CH) [96]. In addition, the following coordination steps had a benefit from MAS. As an example, [^99m^Tc(CO)_3_(OH_2_)_3_]^+^ (Alberto’s reagent, **8**, Figure 4) was reacted with the bifunctional chelating ligand dithiazole valeric acid by comparing three different methods (i.e., microfluidic reactor, MW, and CH). As in the case of the precursor, MAS demonstrated better performances when compared with CH. Labeling of dithiazole valeric acid at low concentrations did not occur using CH (100 °C), whereas the yield after 7.85 min was 18% in the MW reactor. However, the microfluidic reactor outperformed at low concentrations of ligand, resulting in higher yields than MW and CH in all conditions [97].

Less interesting from a coordination chemistry point of view but worthy to be mentioned because of its practical importance is the formulation of ^99m^Tc-antimony trisulphide. In Australia, it is a standard radiotracer for preoperative lymphoscintigraphy, and it can be prepared with a procedure similar to that previously reported (i.e., the addition of ^99m^TcO_4_^−^ and HCl to a vial containing colloidal antimony trisulfide and the heating at 100 °C for 30 min). Additionally, in this case, the MW procedure considerably reduces the heating period (15 s) with a RCP of 99% [98].

An alternative to [M(CO)_3_(OH_2_)_3_]^+^ precursors may be represented by [M(η^5^-C_5_H_5_)(CO)_3_]-containing products. Unfortunately, for the preparation of ^99m^Tc analogues of these compounds there is the need to employ harsh reaction conditions, organic solvents, and other restrictions difficult to be suitable for routine clinical use. As far as carboranes (i.e., polyhedral boranes in which a BH^−^ unit has been formally replaced by an isoelectronic C-H unit) are known as inorganic analogues of aromatic molecules, some groups tried to use them as surrogates for Cp derivatives. Some Tc and Re metallocarboranes (see series **9**, Figure 4) were prepared in aqueous media in a single step with good yield, and as we have learned, MWs can improve the synthesis in terms of speed [99,100,101].

Finally, few other scattered examples of the application of MW irradiation to Re chemistry were reported: (i) the synthesis of dirhenium paddlewheel complexes [102]; (ii) the reaction between [ReBr(CO)_5_] and tripodal nitrogen ligands derived from tris(pyrazolyl)methane [103] or 3-(2-pyridyl)pyrazole [104]; and (iii) the synthesis of three tricarbonyl rhenium(I) pentylcarbonato complexes of the general formula *fac*-[Re(CO)_3_(α-diimine)(pentyl)] and their conversion to carboxylato, sulfonato, and chlorido complexes [105]. Furthermore, [ReCl_3_O(PPh_3_)_2_] was synthesized (together with analogous complexes of Ru(III) and Rh(III)) via MW reflux in EtOH/water in 30 min with a 94% yield instead of 5 h with conventional reflux. In general, it was found that the reaction times for the modified refluxing MW apparatus were higher than those with MW Teflon autoclave but significantly lower than those under conventional reflux [63].

## 4. Late Transition Elements of Groups 8–12

### 4.1. Iron

The discovery of the archetypal metallocene ferrocene ([Fe(η^5^-C_5_H_5_)_2_], bis(η^5^-cyclopentadienyl)iron(II), **10**, Figure 4), in 1951 raised interest in the chemistry of cyclopentadienido anion derivatives to be applied in several fields from medicinal chemistry to catalysis.

In this context, MAS procedures were applied to the synthesis of several sandwich and piano-stool iron complexes.

The synthesis of [Fe(η^6^-arene)(η^5^-cyclopentadienyl)]^+^ compounds (**11**, Figure 4) was carried out by MW heating using a solid CO_2_-cooled system in a commercial MW oven. The use of MW reduced the reaction times of the mixture arene/ferrocene/AlCl_3_/Al from several hours to a few minutes, usually with higher yields with respect to the use of CH. The decomplexation of some of these complexes was also carried out by an MW-assisted procedure using graphite as a very efficient MW absorber [106,107].

Similarly, as already mentioned in Section 3.3, MWs from a domestic oven were used for the synthesis of sterically hindered [Fe(η^6^-arene)(η^5^-cyclopentadienyl)]^+^ complexes with *tert*-butyl substituents from arene, ferrocene, AlCl_3_, and Al; the MAS procedures resulted in products in 9–95% yields in 3.5 min [90].

Other [Fe(η^6^-arene)(η^5^-cyclopentadienyl)]^+^ complexes containing oxygen, nitrogen, and carbonyl substituents were prepared by MW-assisted ligand exchange on ferrocene although the yields were not always optimized. Oxygen-substituted complexes were prepared by ligand exchange reactions using ferrocene, arene, Al, and AlCl_3_ in 1,2,4-trichlorobenzene as solvent in an MW oven in few min. For complexes containing carbonyl substituents, MW irradiation was applied to the mixture of the (η^6^-fluorobenzene)(η^5^-cyclopentadienyl)iron(II) complex with nitroethane and K_2_CO_3_ in dry DMF to obtain first the α-nitroethylbenzene complex (after 60 s) and then the acetophenone complex (after further 60 s in the presence of 2 M HCl). The [(η^6^-fluorobenzene)(η^5^-cyclopentadienyl)iron(II)(1+)] was the starting material also for [(η^6^-diphenylamine)(η^5^-cyclopentadienyl)iron(II)(1+)] complex when reacting with aniline in the presence of Et_3_N, flaked graphite, and dry DMF and applying MW for 5 min. Such a method was also employed for the one-pot synthesis of a *N*-arylated amino acid. Other miscellaneous reactions were reported in the same papers [108,109].

In addition, the synthesis of ferrocenyl-substituted heterocycles (e.g., thiophenes, furans, pyrroles, pyrimidine, and pyrazole) could benefit from the use of an MW oven in obtaining significantly high yields [110].

Chemically and thermally stable Dewar benzene–ferrocene conjugates, synthesized from tetraalkylcyclobutadiene, AlCl_3_, and 3-ferrocenylpropynoates, did not rearrange to their corresponding phenylferrocenes upon heating to their melting points or to 150 °C in DMSO for 30 min. Furthermore, heating to 180 °C in DMSO resulted in their decomposition. On the contrary, with MW heating at 180 °C for 6 h in THF, the rearrangement to phenylferrocenes took place (about 80% yield) [111].

Acetylferrocene (**12**, Figure 4) was condensed with aldehydes in the presence of solid KOH and the ionic liquid Aliquat^®^. In the case of piperonal and paramethoxybenzaldehyde, CH leads to slow reactions (e.g., reaction incomplete after 18 h), whereas with MW irradiation, they were accelerated (few minutes) with good yields. Moreover, ferrocene carboxaldehyde was condensed with ketones under MW heating (few min) to speed up slow traditional procedures [112].

Starting from ferrocene, acetylferrocene was rapidly prepared using MW (300 W, 5 min, 125 °C) in a yield higher than with CH (75% vs. 40–60%). The [Fe(η^5^-C_5_H_5_)(CO)_2_]_2_ dimer was prepared with MW in 88% yield in 10 min (150 °C in DMF) instead of 24–48 h reflux in boiling octane or xylenes. From this compound, piano stool complexes, such as [Fe(η^5^-C_5_H_5_)(CO)_2_I] (150 W, 10 min, 90 °C, 90% yield) and [Fe(η^5^-C_5_H_5_)(CO)I(PPh_3_)] (overall 20 min, 90 °C and 76% yield), were obtained (see series **13**, Figure 4). [Fe(η^5^-C_5_H_5_)(CO)(COMe)(PPh_3_)] was rapidly synthesized in 86% yield from PPh_3_, [Fe(η^5^-C_5_H_5_)(CO)_2_Me], and acetonitrile (300 W, 60 min, 110 °C vs. 48 h traditional reflux). Finally, bisphosphane iron complexes were prepared from K[Fe(CO)_4_H] in 5 min (150 W, 100 °C, 44–67% yield) instead of refluxing for 2–12 h [113].

Two other series of iron(II) piano-stool complexes with bidentate phosphane or mixed phosphorus–nitrogen ligands were prepared upon reaction with [Fe(η^5^-C_5_H_5_)(CO)_2_I] or [Fe(η^5^-C_5_H_5_)(naphthalene)]^+^ under MW irradiation or using flow chemistry. As reported above, the reaction of [Fe(η^5^-C_5_H_5_)(CO)_2_I] with PPh_3_ resulted in complex [Fe(η^5^-C_5_H_5_)(CO)I(PPh_3_)] (THF, 150 W, 130 °C, 6 min, 90% yield), whereas the reaction with PBu_3_ and P(NMe_2_)_3_ gave the cationic species [Fe(η^5^-C_5_H_5_)(CO)_2_(PR_3_)]^+^ (THF, 130 °C, 6 min, 16–43% yield). Under the same conditions, dppe gave the cationic complex [Fe(η^5^-C_5_H_5_)(CO)(dppe)]^+^ (54% yield). The reaction between complex [Fe(η^5^-C_5_H_5_)(η^6^-napthalene)]^+^ and dppe after MW irradiation (40 W, 3.5 min) in THF/CH_3_CN resulted in a cationic acetonitrile complex in 92% yield [114].

The iron carbonyl complex [Fe_2_(CO)_9_] was used as an iron source in the quick and easy MAS of a single-phase LiFePO_4_, employing NH_4_H_2_PO_4_ and CH_3_COOLi (80 °C, 10 min) [115].

Finally, as reported in Section 3.1, the reaction of a *o*-vanillin-based Schiff base ligand with several metal chlorides (including Fe(II)) under MW radiation (8–10 min) resulted in the synthesis of the corresponding complexes [69].

### 4.2. Ruthenium and Osmium

**Figure 5:**
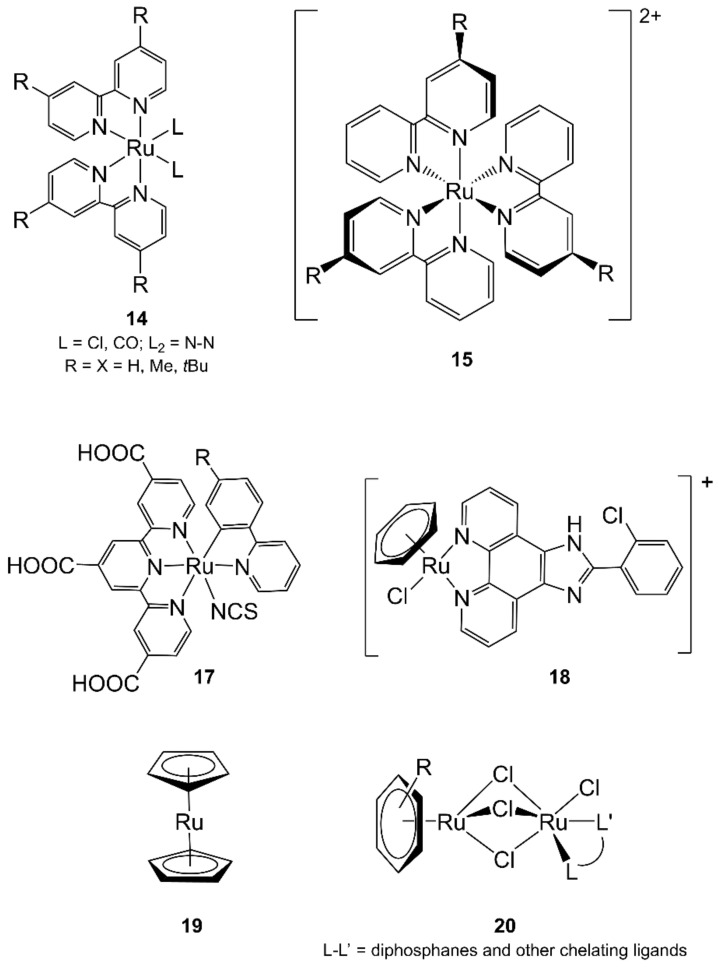
A selection of Ru complexes cited in the text.

Several ruthenium complexes containing 2,2′-bipyridine (bpy), 2,2′:6′,2″-terpyridine (terpy), 1,10-phenanthroline (phen), or their derivatives were prepared with MW irradiation. One of the first examples was the MAS of [Ru(bpy)_2_Cl(CO)]Cl (see series **14**, Figure 5), which was already reported in Section 3.2, together with the synthesis of miscellaneous metal complexes. This Ru complex was prepared in 1 min (instead of 1 week) in 70% yield (Teflon autoclave, 500–650 W) [62].

The same authors reported the use of a thick-walled glass reaction vessel for MAS in a modified MW oven to prepare simple Mo, Ru, and Rh complexes (see Section 3.2). For example, [Ru(bpy)_3_]^2+^ was prepared in 10 min at 133 °C in a 87% yield. In the same paper, [Ru(1,4,7-trithiacyclonane)_2_]^2+^ was prepared in 70 min at 117 °C in 96% yield and [RuCl_2_(cycloheptatriene)]_2_ in 9 min in 66% yield [65].

The α-diimine-Ru(II) complexes [Ru(L-L)_3_][PF_6_]_2_ (L-L = bpy, phen, 4,4′-di-*tert*-butyl-2,2′-bipyridine) and [Ru(terpy)_2_][PF_6_]_2_ were prepared with MW heating in good yields (60–94%). The procedure consisted of two steps of 20 s of MW radiation (650 W), separated by a cooling period of 25 min [116]. Other various α-diimine complexes were rapidly prepared by MW irradiation in a domestic oven with reflux condenser: this method reduced reaction times from 4 h to 20 min with higher yield (60–90%) [117].

More recently, [Ru(bpy)_3_](ClO_4_)_2_ was prepared by reacting RuCl_3_ with bpy in refluxing ethylene glycol for 20 min with N_2_ bubbling under MW irradiation (90% yield). Other Ru(II) polypyridine complexes were prepared with similar procedures with a yield of 65–95% within 20 min [118].

Another series of Ru(II)-bpy of general formula [RuCl_2_(R-bpy)_2_] (R = H, Me, *t*Bu; see series **14**, Figure 5) was synthesized with MAS between the [RuCl_2_(cod)]_n_ polymer (cod = 1,5-cyclooctadiene) and substituted bpy in DMF (microwave setup: 30 s, 600 W followed by 45 min, 200 W). The final complexes were rapidly isolated (ca 1–2 h instead of refluxing DMF for 10–72 h) in at least 87% yield and high purity from the reaction mixture. Further MAS reactions of [RuCl_2_(R-bpy)_2_] with substituted ligands N–N (i.e., benzimidazoles, phen or bipyrimidine) in DMF/water mixtures and similar microwave setup resulted in the formation of mixed ligand complexes [Ru(N–N)(R-bpy)_2_]Cl_2_ (see series **14**, Figure 5) without the formation of side products (differently from thermal conditions) [119].

[Ru(terpy)_2_][PF_6_]_2_ was also prepared from RuCl_3_ and terpy in refluxing ethylene glycol for 4 min in an MW oven (325 W) in 89% yield (vs. 21–65% in 3–4 h refluxing DMF or EtOH). In the same paper, Ru(II) and Rh(III) complexes with chiral terpy ligands, [RuL_2_][PF_6_]_2_ (L = dipineno-[5,6:5″,6″]-fused terpy, or dipineno-[4,5:4″,5″]-fused terpy) were prepared in good purity and yields with MAS procedures in ethylene glycol (4 min, 375 W) from RuCl_3_ [120].

The traditional two-step reaction of RuCl_3_ with 4′-chloroterpyridine was improved by using MW heating. It was complete in 5 min (instead of refluxing for at least 1 h) and gave [Ru(4′-chloro-terpy)_2_][PF_6_]_2_ in about 90% yield [121].

A rare triple helicate [Ru_2_L_3_]^4+^ (L = 5,5′′′-dimethyl-2,2′:5′,5′′:2′′,2′′′-quaterpyridine) was synthesized from RuCl_3_ in final 36% yield upon MW heating in dry, degassed ethylene glycol for 4.5 h (65 % of 400 W in a pressure vessel at 225  °C). On the contrary, the attempts to react RuCl_3_ and L in a 2:3 ratio in EtOH under reflux for two weeks resulted in the production of a complex mixture of products, including polymeric material [122].

The MAS was applied to reactions involving structurally more complicated α-diimine complexes. The [Ru(dcbpy)L_2_]^2+^ (dcbpy = dicarboxybipyridine; L = pyrrole- and pyrrolidine-containing bpy) complexes were prepared by a two-step procedure: RuCl_3_ reacted with an excess of L under MW irradiation in DMF at 160 °C for 8 min (instead of conventional 12 h), and then, the resulting chlorido ligands were substituted by dcbpy upon refluxing acetic acid, resulting in the final complexes with a good overall yield (60–76%) [123].

Complexes of the type [RuL_3_][PF_6_]_2_ containing 4-alkoxycarbonyl-substituted unsymmetrical bpy ligands (L) were prepared by reaction of L with RuCl_3_ in ethylene glycol in the presence of *N*-ethylmorpholine under MW irradiation (250 W, 200 °C, 4 min instead of refluxing 12–14 h). With this procedure, exclusively *fac* isomers (**15**, Figure 5) were obtained [124].

In the synthesis of some [Ru(dcmb)_3–*n*_(tbbpy)*_n_*][PF_6_]_2_ complexes (*n* = 0–3, dcmb = 4,4′-dimethoxycarbonyl-2,2′-bipyridine, tbbpy = 4,4′-di-*tert*-butyl-2,2′-bipyridine), [RuCl_2_(dcmb)_2_] was obtained by using MW irradiation in dry DMF with two equivalents of dcmb and [RuCl_2_(cod)]_n_ in 1 h (MW setup: 30 s, 600 W followed by 60 min, 200 W, 90% yield vs. 50 h thermal reaction, 78% yield) [125].

MW heating was used to prepare highly crowded [RuL_3_]^2+^ and [Ru(L)(bpy)_2_]^2+^ (L = 3,3′-dimethylene-2,2′-bibenzo[g]quinoline or bisbenzo [2,3:9,8]-1,10-phenanthroline) complexes from RuCl_3_ and [Ru(bpy)_2_Cl_2_]. The reactions of such highly sterically encumbering ligands resulted in only the recovery of unreacted materials when refluxing aqueous EtOH. On the contrary, when heated in an MW oven in ethylene glycol, the complexes were obtained in 15–30 min with 15–44% yields [126].

**Figure 6:**
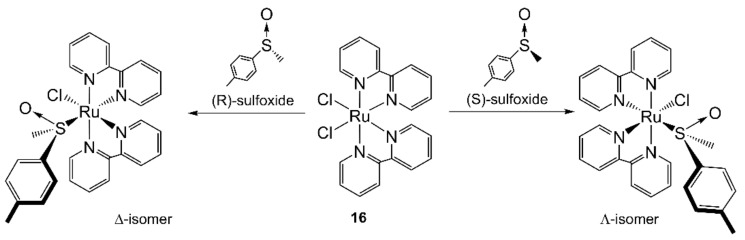
Scheme of the reaction to obtain ruthenium bis(α-diimine) sulfoxide complexes adapted from [127].

Ruthenium bis(α-diimine) sulfoxide complexes were prepared after MW irradiation of racemic *cis*-[RuCl_2_L_2_] (L = bpy or phen, **16**, Figure 6) and (*R*)- or (*S*)-methyl *p*-tolyl sulfoxide [127]. The substitution of one chloride by chiral sulfoxide on *cis*-[Ru(bpy)_2_(Cl)_2_] would lead to the formation of Λ and Δ isomers *cis*-[Ru(bpy)_2_Cl(dmso)]^+^ in 1:1 ratio. Reaction of *cis*-[Ru(bpy)_2_Cl_2_] with enantiomerically pure (*R*)-sulfoxide (MW, 2 min at 375 W) resulted in full conversion and a slight increase in the diastereoisomeric excess (de) of the Δ isomer (74% de with MW instead of 68% de with CH). On the contrary, the MW reaction with the (*S*)-sulfoxide gave a slight increase of the Λ isomer. The reactions performed with *cis*-[RuCl_2_(phen)_2_] paralleled those mentioned above, but 4 min of MW irradiation was necessary instead of 2 min.

Cyclometallated ruthenium(II) complexes [Ru(C^N)(NCS)(tcterpy)] (C^N = 2-phenylpyridinato or 2-(4-(2-phenylethynyl)phenyl)pyridinato; tcterpy = 4,4′,4″-tricarboxy-2′,6′-terpyridine, **17**, Figure 5) were prepared refluxing the intermediates, obtained from the reaction of [RuCl_3_(Me_3_tcterpy)] with C^N and ammonium thiocyanate, for 10 min in an MW synthesizer, followed by further 24 h refluxing in the presence of triethylamine. The relative position of the pyridyl of C^N as well as of the central pyridyl of tcterpy gives rise to different isomers, but the use of MW allowed the formation of only one of them [128].

As reported for rhenium compounds (see Section 3.4), the ligand 3-(2-pyridyl)pyrazole (pypzH) was used to prepare Re(I) and Ru(II) complexes. In particular, a mixture of [Ru(bpy)_2_Cl_2_], pypzH and NH_4_PF_6_ in water was heated in an MW oven for 10 min at 150 °C (yield 82%). Moreover, [Ru(bpy)_2_Cl_2_] was obtained in 78% yield after heating a mixture of [RuCl_2_(dmso)_4_] and bpy in CHCl_3_ in an MW oven for 1 h at 150 °C (vs. 8 h in refluxing DMF) [104].

MW heating at 200 °C for 20 min was used to synthesize *mer*-[Ru(dqp)_2_]^2+^-based (dqp = 2,6-di(quinolin-8-yl)pyridine) complexes in high yield (49−87%). When the temperature was lowered to 180 °C, the formation of *cis* and *trans fac*-isomers (56% and 12% yields, respectively) occurred after 5 min of reaction. The microwaves also allowed the synthesis of the dqp ligands and a dinuclear complex by Suzuki coupling (80 °C, 2 h, 78% yield) [129].

Tris-chelated ruthenium(II)-arylazoimidazoles complexes [Ru(*o/p*-RaaiR′)_3_]Cl_2_ (RaaiR′ = 1-alkyl-(2-arylazo)imidazole, R= H, *p-*Me, *p-*OMe, o-OMe, R′ = Me, Et, CH_2_Ph) were prepared by heating RuCl_3_, dry EtOH, and then 1-alkyl-(2-arylazo)imidazole in two steps to obtain the final complexes with a yield of 55–70% (Teflon reactor in MW oven, 450 W, three lots of 5 min with 5 min interval each step) [130].

MW and CH in different solvents were compared in the synthesis of ruthenium (and also nickel—see Section 4.6) complexes containing the 9-anthracene carboxylate ligand (9-atc). The diruthenium compound [Ru_2_(9-atc)_4_Cl] was prepared in a Teflon vessel starting from [Ru_2_Cl(μ-O_2_CMe)_4_] and 9-atc in EtOH and heating in MW oven for 16 h at 100 °C (final yield 56%). In this case, MW significantly underperformed with respect to CH as far as traditional reflux in MeOH/H_2_O for 4 h resulted in 70% yield [131].

The metal–metal bond paddlewheel Ru(II) compounds tetrakis(diaryltriazenido)diruthenium(II) were prepared under MW at 130 °C in EtOH (vs. multistep CH in 2 d) in good yield (78% in 2 h or 90% in 8 h instead of 44% with CH) starting from a mixture of chloridotetrakis(acetato)diruthenium(II,III), 1,3-diphenyltriazene, or 1,3-di(*p-*fluorophenyl)triazene) in the presence of triethylamine [132].

The reduction of Ru(IV) to Ru(III) in the kinetically inert (µ-oxo)bis(pentachlororuthenate) ion, [Ru_2_Cl_10_O]^4–^, was studied in aqueous acidic alcohol solutions with both MW and CH. The reaction time with MW heating (up to 30 min at 98 °C) was reduced of one order of magnitude in comparison with CH [133].

Finally, MAS of the osmium complex [Os_2_Cl_3_(PEt_2_Ph)_6_]Cl from (NH_4_)_2_[OsCl_6_] and diethylphenylphosphane (PEt_2_Ph) was carried out in an MW reactor within 5 min at 150 °C (60% yield) rather than refluxing in aqueous EtOH for approximately one week [134].

Moving to organometallic compounds, piano-stool complexes of Ru(II) with η^6^-arene units or, more generally, with aromatic ligands are known for their diverse and peculiar catalytic activities. In a completely different field, the half-sandwich compounds of Ru(II) showed interesting anticancer activity. Therefore, the synthesis of this kind of compounds was extensively studied, and MW irradiation was also exploited.

Starting from [Ru(η^5−^C_5_H_5_)Cl(PPh_3_)_2_], the synthesis of a bis(triphenylphosphane)thiolato [Ru(η^5−^C_5_H_5_)(PPh_3_)_2_(SPh)] complex was performed under MW conditions in a focused MW reactor (2 h). While the yield was high with CH, under MW irradiation, a mixture of at least five compounds was formed, and the yield of the desired complex was 20% (MW conditions: 100 W, 60 s, in diethylene glycol). Therefore, two PPh_3_ were substituted with one methylenebis(diphenylphosphane) (dppm), and the corresponding (more stable) [Ru(η^5−^C_5_H_5_)(dppm)(SR)] compounds were prepared in higher yield under MW heating (90–120 s) [135].

One of the first examples of MAS applied to Ru(II)–arene compounds was already mentioned in Section 3.4. The synthesis of [RuCl_2_(η^6^-C_6_H_6_)]_2_ and [RuCl_2_(η^6^-cymene)]_2_ was performed starting from RuCl_3_ and the ligands under MW reflux in MeOH or EtOH, giving the product in 30 min (10 min with cymene ligand) with 85% yield (67% with cymene ligand) instead of 3–4 h with conventional reflux. The use of a Teflon autoclave further reduced the reaction time (<1 min) [63].

The arene Ru(II) complex [Ru[(η^6^-C_6_H_6_)(*o*-ClPIP)Cl]Cl (*o*-ClPIP = 2-(2-chlorophenyl)-1*H*-imidazo [4,5-*f*][1,10]phenanthroline, **18**, Figure 5) was prepared with MAS heating of [Ru(η^6^-C_6_H_6_)Cl_2_]_2_ and *o*-ClPIP in dichloromethane at 60 °C for 30 min to obtain the product in higher yield (91%) than with traditional procedures [136].

As in the case of ferrocene, ruthenocene [Ru(η^5^-C_5_H_5_)_2_] (**19**, Figure 5) can also exchange one of its ligands. Cationic [Ru(η^6^-arene)(η^5^-C_5_H_5_)]^+^ complexes were obtained from a mixture of ruthenocene, arene, Al, AlCl_3_, decalin, and TiCl_4_. The mixture was stirred for 5 min before heating for 15 min at 230 °C with MW (vs. CH at 140 °C for 3 d), and the final complexes were isolated in moderate to excellent yields. Activated [Ru(η^5−^C_5_H_5_)(CH_3_CN)_3_][PF_6_] was the starting material for another series of Ru(II)-arene complexes containing naphthoquinone, tetralindione, 1,4-dihydroxynaphthalene, and 1,4-dimethoxynaphthalene. For example, [Ru(η^5^-C_5_H_5_)(η^6^-5,8-naphthoquinone)][PF_6_] was prepared with MW irradiation at 100 °C for 30 min (80% yield) [137].

The MW heating was applied to substitution reactions on [RuCl_2_(η^6^-*p-*cymene)]_2_ or [Ru(η^6^-1,3,5-C_6_H_3_iPr_3_)Cl_2_]_2_ complexes with chelating ligands L-L′, such as chelating diphosphanes, a bulky α-diimine, a chiral *P*–*N*-, a non-chiral *P*–*N*-, and *P*–*S*-chelates. The reactions of the starting [Ru(η^6^-arene)Cl_2_]_2_ with L-L′ resulted in dinuclear complexes [(η^6^-arene)Ru(μ-Cl)_3_RuCl(L–L′)] (**20**, Figure 5) in moderate to good yield (up to 91%) when heated in an MW reactor for 4 h at 130–150 °C in THF. Depending on the experimental conditions, the yields with MW can be higher or lower with respect to CH. It is interesting to note that complexes with *P*–*S*- and *P*–*N*-chelate ligands are chiral (stereogenic metal center), but the compound containing the chiral (*R*)-(–)-2-[2-(diphenylphosphanyl)phenyl]-4-phenyl-2-oxazoline ligand was formed in a highly diastereoselective way [138].

The reactivity of Ru and Os carbonyl species has been studied for decades with the aim to demonstrate that discrete metal clusters may serve, to a first approximation, as models of metal surfaces in chemisorption and catalytic processes. The low reactivity of such M(0) species led to an extensive search for methods to activate them.

Using a gas-loading accessory, [Ru_3_(CO)_12_] (110 °C, 10 min), [H_4_Ru_4_(CO)_12_] (130 °C, 15 min), and [H_2_Os_3_(CO)_10_] (150 °C, 15 min) were prepared in high yields using MW heating. In the case of [Ru_3_(CO)_12_], the substitution of the ligands with triphenylphosphane (100 °C, 5 min) or phenylacetylene (1 min, 110 °C) was also carried out in an MW reactor with excellent yield [139].

**Figure 7:**
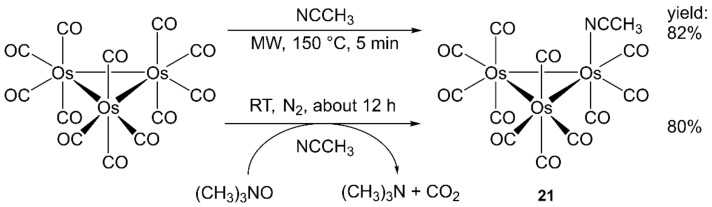
Scheme of the reaction to obtain the activated [Os_3_(CO)_11_(NCCH_3_)] intermediate.

Much of the chemistry of Ru and Os trimetallic carbonyls went through the lightly stabilized clusters [M_3_(CO)_12-n_(NCCH_3_)_n_] (*n* = 1 or 2; M = Ru, Os,) that can be prepared by the reaction of [M_3_(CO)_12_] with trimethylamine *N*-oxide (CH_3_)_3_NO in the presence of CH_3_CN. This procedure provides the desired products in very good yield and purity but requires the exclusion of air and moisture and takes over many hours. The CO substitution reaction was carried out on [Os_3_(CO)_12_] in acetonitrile with MW heating; [Os_3_(CO)_11_(NCCH_3_)] (**21**, Figure 7) was obtained after 5 min in 82% yield without the use of the decarbonylation reagent (CH_3_)_3_NO.

When the activated clusters meet other donor ligands L in solution, they easily exchange CH_3_CN with L. This reaction was further accelerated by MW irradiation and without the need to separate the activated intermediate. Actually, [Os_3_(CO)_12_] and acetonitrile were irradiated in the MW reactor (200 W, 150 °C, 5 min), and then, the solvent was evaporated, and pyridine or PPh_3_ in dichloromethane was added and further irradiated (100 W, 47 °C for 2 min in the case of pyridine or 23 °C, 1 h in the case of PPh_3_). [Os_3_(CO)_11_(pyridine)] and [Os_3_(CO)_11_(PPh_3_)] were produced in 67% and 80% yield, respectively [140].

### 4.3. Cobalt

A series of papers reported on the synthesis of complexes with Schiff bases and Co(II) as well as Ni(II), Cu(II), and other metal ions. The ligands were derived from salicylaldehydes, benzaldehydes, naphthaldehydes, and thiophene-2-carbaldehyde with anilines, phenylenediamines, pyridines, and thiazoles. The reactions were carried out using both CH (in refluxing EtOH containing the metal salts and the Schiff bases) and MW conditions (in open glass vessels containing ethanolic mixtures of the metal salts and the Schiff base; irradiation power of 800 W). The reaction gave ML_n_ complexes (*n* = 1 or 2 depending on the M:L ratio employed, 1:1 or 1:2), and under MW conditions, it was completed in a shorter time (4–9 min vs. >3 h) with higher yields (approximately 80–87% vs. about 60–70%) [71,141,142,143,144,145,146,147,148].

Two other papers containing the MAS of several transition metal complexes, including Co(II), with Schiff bases were reported in Section 3.2 and Section 3.3 [69,70,86].

The reaction of cyclohexylphosphonic acid with CoSO_4_ in water by using a variety of synthetic strategies produced exclusively [Co(cyclohexylphosphonate)(H_2_O)]_n_, but the reaction periods varied considerably for different methodologies. As expected, the irradiation of the reactants in an MW oven (100 W) required only a few minutes for the isolation of the product in nearly quantitative yields, whereas the hydrothermal and room-temperature syntheses required a few days for the completion of the reaction [149].

The reaction of CoCl_2_ with *N*,*N*-*bis*(2-hydroxyethyl)glycine (H_3_bic = bicine) and NEt_3_ in EtOH under solvothermal conditions (140 °C, 72 h) resulted in a mixture of complexes [CoCl(H_2_bic)] and [Co_9_(bic)_2_Cl_4_(Hbic)_4_]_2_. However, by using MWs (140 °C, power: 150 W and pressure: 20.4 atm for a total of 15 min), the selectivity improved: the monocobalt complex was selectively obtained with a CoCl_2_:NEt_3_ ratio of 1:0.13, whereas the [Co_9_] can be isolated in 1:1 ratio [150].

In the MAS of phthalocyanine Co(II) complexes (but also Ni(II), Cu(II), and Zn(II)), a relationship between yield and maximum temperature reached by MW irradiation (connected to the type of salt used in the synthesis) was observed. The reaction time was 24 h for the synthesis in refluxing 1-hexanol using oil-bath heating but only 10–15 min for the MAS in glycerin (open vessel, domestic oven). In this case, the MW did not always give higher yields [151].

**Figure 8:**
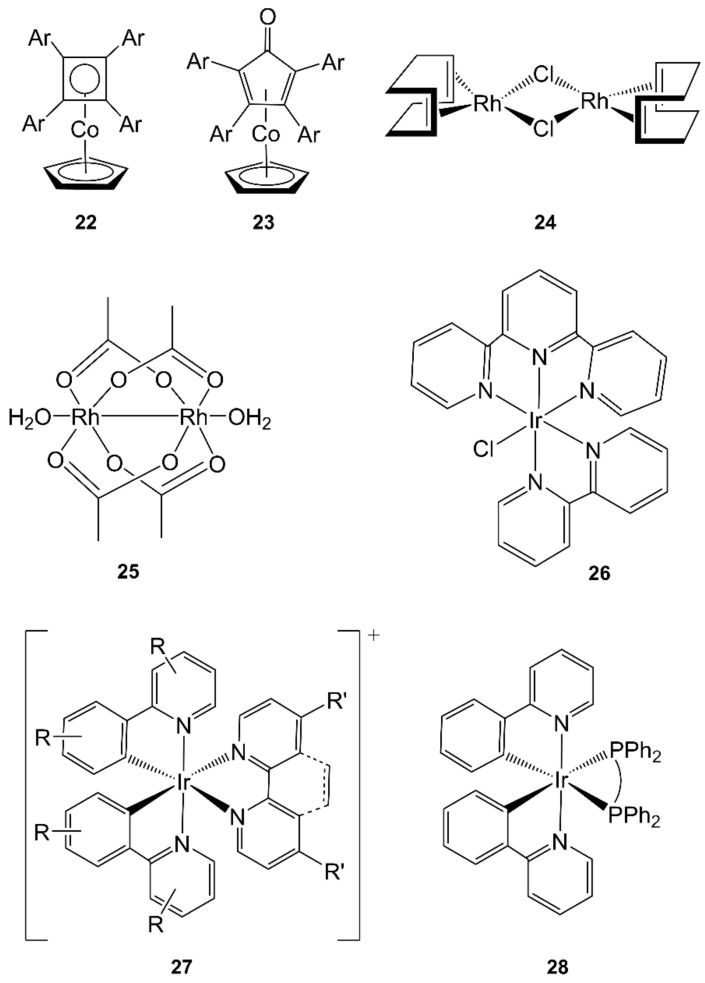
A selection of complexes containing transition metals of group 9 cited in the text.

Finally, MW heating in sealed tube of diaryl acetylenes with [Co(η^5^-C_5_H_5_)(CO)_2_] in *p-*xylene (175 °C, 10 min, maximum power = 50 W) provided access to metallocenes in both the cyclobutadiene [Co(η^4^-Ar_4_C_4_)(η^5^-C_5_H_5_)] (**22**, Figure 8) and cyclopentadienone [Co{(η^4^-Ar_4_C_4_(CO)}(η^5^-C_5_H_5_)] families (Ar = arene; **23**, Figure 8). Detailed examinations of heating approaches showed that reactant concentrations in MAS were higher than those of the corresponding conventional reactions, and simultaneously, both temperature and pressure were significantly enhanced. The reaction outcomes results did not indicate the existence of a specific MW effect, so the performance of MAS resulted in the combination of these effects [152].

### 4.4. Rhodium

Historically, the synthesis of Rh(III) (but also Mo, Ru, and Re) complexes was employed to evaluate the advantages of the (new at that time) “microwave dielectric loss heating effect” over the conventional reflux. In a continuous improvement process [61], [RhCl_2_(cod)]_2_ (**24**, Figure 8) was obtained from RhCl_3_ and 1,5-cycloctadiene in EtOH/water mixtures in 25 min in an open MW system instead of 18 h CH and in less than 1 min in a more expensive MW Teflon autoclave (250–350 W power) [63]. A thick-walled glass reaction vessel specifically designed for an MW oven further improved the synthesis (see Section 3.2) [65].

The synthesis of *cis*-[Rh(bpy)_2_X_2_][PF_6_] (X = Cl, Br, I) in ethylene glycol by both MW and CH resulted in clean and rapid reactions (1.25–4 min vs. 20–65 min) with high but similar yields. The MW method utilized a domestic MW oven without modifications and common laboratory glassware; for this reason, the temperature was harder to control [153].

Binuclear rhodium(II) tetraacetate [Rh_2_(CH_3_COO)_4_(H_2_O)_2_] (**25**, Figure 8) was obtained under the action of MW on a mixture of RhCl_3_, CH_3_COOH, and EtOH in closed autoclaves irradiated for 5−15 min at 100–150 °C in the thermostatic mode. The yield of the desired complexes increased with the concentrations of CH_3_COOH and EtOH to a value beyond which no further growth was observed. Both the temperature and the reaction time increased the yield, too; however, in these cases, temperatures >140 °C and heating time above 10 min were detrimental to the yield, probably due to some decomposition. Under the optimal conditions, the yield was close to 100%, whereas for CH, it was no higher than 75% [154].

Finally, the attachment of ^211^At^−^, ^131^I^−^, and ^125^I^−^ to Rh(III) and Ir(III) complexed with the macrocyclic crown thioether 1,5,9,13-tetrathiacyclohexadecane-3,11-diol at nanomolar concentrations was studied. The complexes labeled with ^211^At (a short-lived α-emitting isotope with a half-life of 7.2 h), after appropriate purification, could be used as precursors for the labeling of biomolecules such as monoclonal antibodies. The use of MW instead of CH reduced the reaction time from 1–1.5 h to about 20–35 min with an approximate yield of 80%, limiting the loss of the radiotracer by spontaneous decay [155].

### 4.5. Iridium

Ir(III) is often considered to be characterized by a great inertness of the coordination sphere, requiring harsh reaction conditions to react. For this reason, MW can represent a way to speed up Ir(III) chemistry.

The most abundant examples of the application of MAS to Ir compounds concern the use of *N*-heterocyclic ligands. For example, tris(2-phenyl-1-quinoline)iridium(III) for electrophosphorescent devices was obtained in 30 min MW irradiation, a time that is 1/20 of that under CH [156].

Several polypyridyl complexes of general formulas [IrCl_2_L_2_]^+^ and [IrCl(L)(terpy)]^2+^ (L = bipyridines, phenanthrolines, pyrazine derivatives, **26**, Figure 8) were prepared by sequential ligand replacement, which occurred in refluxing ethylene glycol in 15 min using an MW oven (500 W) and a round-bottomed flask fitted with a reflux condenser (30–65% yields) [157,158,159].

Two consecutive MW irradiation steps in the same reactor vial were used to synthesize heteroleptic orthometallated iridium(III) polypyridyl photosensitizers [Ir(L)(L′)]^+^ (L = phenylpyridines; L′ = bipyridines, **27**, Figure 8) in good yield, reducing the reaction time from 30 h (IrCl_3_ + phenylpyridines, 12–15 h then bipyridines 15 h, at 120–150 °C in ethylene glycol) to 1 h (IrCl_3_ + phenylpyridines, 50 min then bipyridines 30 min, at 200 °C in ethylene glycol) [160]. A similar reaction scheme was used to synthesize the greenish-blue light-emitting [Ir(ppy)_2_(L)] orthometallated complexes (ppy = 2-phenylpyridine; L = chelating diphosphanes, **28**, Figure 8) in 2-ethoxyethanol with the usual decrease in reaction times (from 12–24 h to 15–30 min) ([161,162]).

Finally, other examples of the application of MAS to Ir complexes were reported in the previous sections [61,62,155].

### 4.6. Nickel

Nickel was frequently used together with other metals in previously cited works. In particular, several Ni complexes containing Schiff base ligands were successfully prepared with the use of MW radiation. Moreover, the ligands themselves were often synthesized exploiting MAS. General considerations can be drawn for the syntheses of the final complexes: the reaction time decreased from hours of CH to minutes, and the yields improved from 60–80% to 80–90%. The use of solvent can also be minimized.

**Figure 9:**
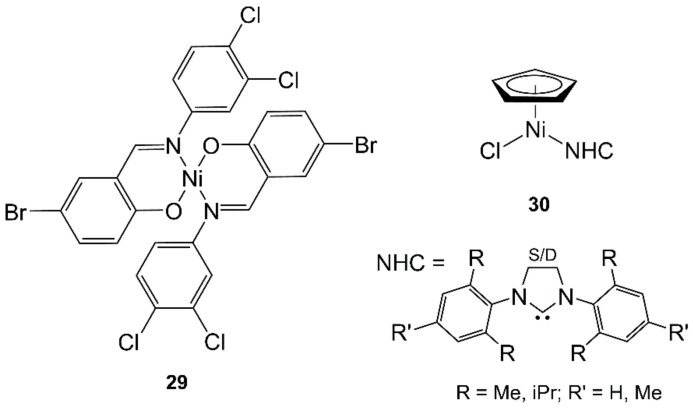
A selection of Ni complexes cited in the text.

The Schiff bases that have been used as ligands for Ni(II) in MAS were obtained from benzaldehyde derivatives, including salicylaldehyde and *o*-vanillin, as the source of the carbonyl group and various classes of organic molecules as the source of the amino group (see Section 3.1, Section 3.2, Section 3.3 and Section 4.3 for details and **29**, Figure 9, as an example) [69,70,71,86,141,142,143,144,145,146,148,163]).

In addition to the above-mentioned examples, a Schiff base trinuclear nickel cluster was synthesized from Ni(ClO_4_)_2_, 2-hydroxybenzaldehyde and aqueous methylamine in acetonitrile/MeOH with MW irradiation for 29 min to obtain [Ni_3_(CH_3_CN)(mimp)_5_]ClO_4_ (mimp = 2-methyliminomethylphenolate) in 87% yield [164].

Other polynuclear Ni(II) complexes were prepared with solvent-free MW heating (150 W, 150 °C, 10 min) applied to Ni(OH)_2_ and 6-chloro-2-hydroxypyridine (chp) resulting in [Ni_7_(chp)_12_(OH)_2_(CH_3_OH)_6_] in low yield (8%). This procedure was applied with moderate yields to the synthesis of two new trinuclear Ni(II) complexes, [Ni_3_(chp)_4_(tBuSALOH)_2_(MeOH)_5_] (tBuSALOH = 3,5-di-*tert*-butylsalycilate) (44% yield at 170 °C for 10 min) and [Ni_3_(chp)_4_(iPrSALOH)_2_(MeOH)_6_] (iPrSALOH = 3,5-di-isopropyl-salycilate) (27% yield at 150 °C for 10 min) [165].

Moreover, as reported in Section 4.2, MW and CH in different solvents were compared in the synthesis of dinuclear Ni complexes containing 9-anthracene carboxylate ligand (9-atc) [131]. The reaction of NiCO_3_·2Ni(OH)_2_, 9-atc and pyridine in a 1:6:12 stoichiometric ratio gave complex [Ni_2_(9-atc)_4_(OH_2_)(py)_4_]·2H_2_O in 56% yield after (i) 20 min heating ramped up to 150 °C and (ii) 2 h isotherm at 150 °C in a Teflon vessel in an MW oven. In this case, conventional stirring at room temperature was more efficient (82% yield in 5 min).

A domestic MW oven was used for the synthesis of both the ligand and the Ni(II) complex obtained from 2′,4′-dihydroxy 4-fluoro chalcone oxime, where Ni(II) is coordinated to the ligand through the phenolic-*O* and azomethine-*N*. The complex was synthesized in 80% yield in 3–4 min at 200 W [166].

Nickel(II) complexes containing 2-amino-6-methylpyrimidine-4-ol and amino acids were synthesized by conventional and MW methods. The MW method resulted to be more efficient than the CH since the preparation time was shorter (4–7 min vs. 45 min), with very high yield (90%). The authors concluded that the MAS was “easier, convenient and eco-friendly” [167].

[Ni(η^5^-C_5_H_5_)Cl(NHC)] complexes (NHC = *N*-heterocyclic carbenes, **30**, Figure 9) were synthesized using MW heating in shorter times (5 or 30 min at 110 °C) and yields higher than or comparable to (about 80%) those of conventional procedures (refluxing THF from 0.5 h to overnight) [168].

**Figure 10:**
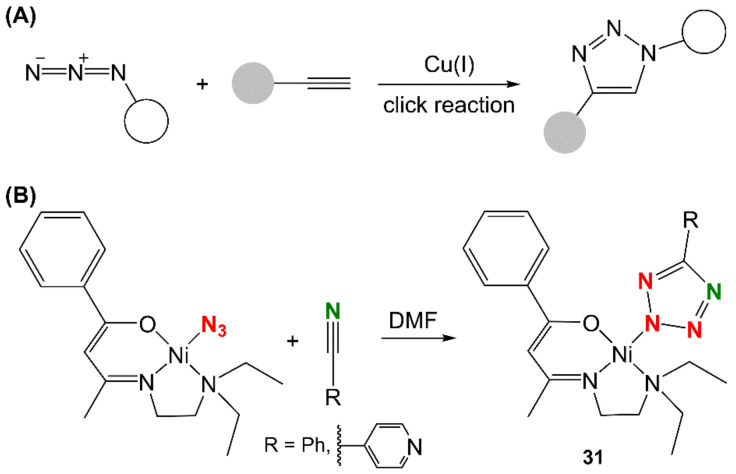
(**A**) The traditional copper-catalyzed azide–alkyne cycloaddition and (**B**) the general scheme of synthesis of nickel tetrazolato complexes adapted from [170].

Click chemistry is one of the most powerful tools for the fast and efficient covalent conjugation of two “partners”. The copper-catalyzed azide–alkyne cycloaddition (CuAAC) is still the most widely used among click reactions because it is typically carried out in the presence of air and/or water and because of the facile modification and incorporation of the necessary reacting groups within biological scaffolds (Figure 10A). Ideally, click reactions would produce quantitatively isolable products in a few minutes at room temperature. However, this wish often clashes with the hard chemical reality, and one must assist the reaction with external energy. For this reason, MW irradiation has earned a place of honor in the field due to the outstanding results achieved by performing CuAAC as a MAS [169]. A particular example of click chemistry is represented by the synthesis of nickel tetrazolato complexes [Ni(L)(5-phenyltetrazolato)] and [Ni(L)(5-(4-pyridyl)tetrazolato)] [HL = 3-(2-diethylaminoethylimino)-1-phenyl-butan-1-one] (**31**, Figure 10B). The compounds were synthesized by MW irradiation (2 h, 130 °C, 60 and 70% yields, respectively), starting from [NiL(N_3_)] exploiting a 1,3-dipolar cycloaddition between azide and organonitriles. What is unique about this reaction is that the azide is coordinated to the metal ion, and it is not part of an organic ligand [170].

Scattered examples of other MAS involving Ni complexes were reported in Section 3.3 and Section 4.3 (i.e., a Ni(II) complex containing a luminol derivative as a tridentate ligand [147], a Ni(II)-phthalocyanine compound [151], and a bimetallic Mn-Ni complex with 3,5-di-*tert*-butylsalicylic acid and 3-dimethylamino-1-propanol [89]).

### 4.7. Palladium

MW-assisted syntheses of Pd compounds received less attention than those involving Ni and Pt complexes even though they belong to the same group of the periodic table. For this reason, the application of MWs was less systematic and more sporadic.

The treatment of diazidopalladium(II) complexes with organonitriles resulted in bis(tetrazolato)-Pd(II) complexes via cycloaddition. The use of MWs accelerated reactions from 12 h (CH reflux) to 1 h (MW) [171].

**Figure 11:**
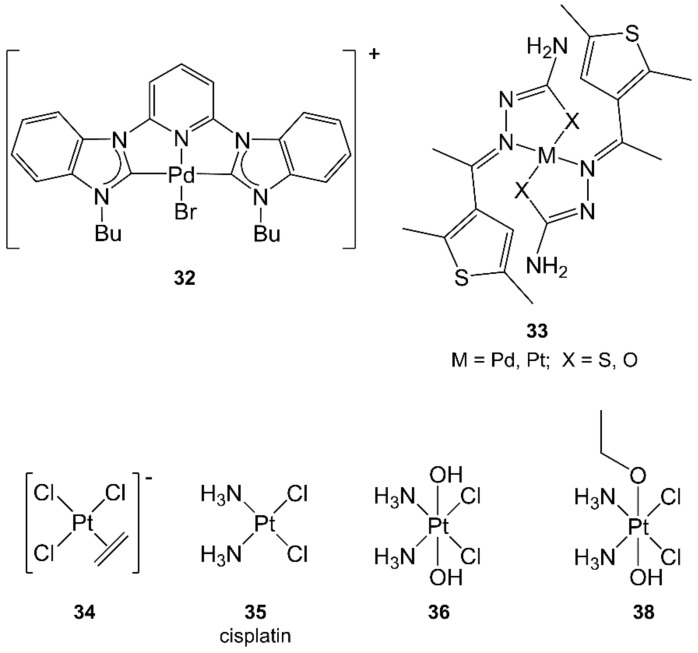
A selection of Pd and Pt complexes cited in the text.

Some pincer palladium complexes were prepared exploiting MWs. A pyridine-bridged bis(benzimidazolylidene) pincer Pd(II) complex (**32**, Figure 11) was easily obtained (25 min, 160 °C) with moderate yield by MW-assisted reaction between diacetatopalladium(II) and the ligand [172]. A NCN-pincer Pd(II) complex containing bulky diphenylhydroxymethyl pyrrolidinyl moieties was obtained by reacting a Pd precursor with the ligand under MW irradiation for 10 min (95 °C) with a yield of 79%, thus with a higher yield and lower time than with CH [173].

The reaction times for the synthesis of palladium(II) complexes containing NHC could be drastically reduced with MW-assisted procedures. Complexes containing acetylacetonate traditionally required refluxing of NHC·HCl salts with palladium(II) acetylacetonate in dioxane for 14–44 h. Using MWs, the products were obtained in high yields after 30 min of heating at 110 °C in THF. Complexes containing 3-chloropyridine, conventionally prepared by heating NHC·HCl with PdCl_2_, K_2_CO_3_, and 3-chloropyridine at 80 °C for 16 h, were obtained in high yields with MW heating at 200 °C after 45 min [174].

Sometimes, Pd and Pt complexes are studied together. For example, solid-state cyclometalation of Pd(II) and Pt(II) complexes containing 1-methyl-2,4′-bipyridinium was assisted by MW irradiation. Multimode irradiation was compared to single-mode resonance irradiation: with a commercial oven, the reaction was carried out in a vermiculite bath only, whereas in single-mode resonance cavity, the energy was concentrated on a small sample, resulting in rapid and quantitative cyclometalation [175].

Finally, MAS of Pd(II) and Pt(II) complexes with 3-acetyl-2,5-dimethylthiophene thiosemicarbazone and 3-acetyl-2,5-dimethylthiophene semicarbazone (**33**, Figure 11) resulted in lower reaction times (min vs. h), lower solvent consumption, and generally higher yield with respect to the CH method [176].

### 4.8. Platinum

Platinum compounds have been widely studied for their applications in different fields, and several Pt(II) and Pt(IV) complexes were prepared using MAS.

First, the MAS of [PtCl(terpy)]Cl from K_2_[PtCl_4_] and terpy in water was reported in the repeatedly mentioned reference [62] (reaction was performed in 1 min vs. conventional 24–100 h, in 47% yield; see Section 3.2).

More recently, polypyridines were used as ligands in the self-assembly of Pt(II) metallacycles with MW-assisted heating, obtaining the products in high purity and high yields within 3–4 h (vs. 4–10 d of CH) [177].

Other Pt(II) complexes containing several pyridines were synthesized starting from [PtCl_4_]^2−^ or [PtCl_2_(cod)] even though not all the procedures overcome the traditional methods [178]. Starting from those results, [PtCl_4_]^2−^ also reacted with phen under MW irradiation (EtOH/water, 60 °C, 5 + 15 min, 10 W) in 51% yield [179].

Cycloplatinated complexes with substituted pyridines were obtained with efficient, ultrafast, MW-assisted syntheses. Microwaves accelerated the synthesis from 1–2 d to 1–6 min but with yields that were not always comparable to those of traditional procedures. Working with irradiation/external cooling cycles of a few minutes allowed temperature and power control and less degradation of Pt(II) reagents and products [180]. Other examples of MW-assisted cyclometallation include those containing 1-methyl-2,4′-bipyridinium [175] and *m*-di(2-pyridinyl)benzene [181] as ligands.

Leadbeater et al. used MAS to prepare well-known Pt(II) complexes [182,183]. First, they synthesized the historical Zeise salt, K[Pt(C_2_H_2_)Cl_3_] (**34**, Figure 11); the reaction was complete with high yield after 15 min at 130 °C using K_2_PtCl_4_ and gaseous ethene in a 1:1:1 water:EtOH:concentrated HCl mixture. This represented a significant improvement with respect to longer (7–14 d) or catalyzed procedures, leading the latter to problems in product isolation [182].

MW heating was applied by both Leadbeater and Hoeschele to the synthesis of cisplatin (*cis*-[PtCl_2_(NH_3_)_2_]) (**35**, Figure 11), the prototype of metal-based anticancer drugs [183,184]. Starting from K_2_[PtCl_4_], KCl, and ammonium salts, cisplatin was obtained after 15 min at 100 °C with yields of 47–74%. Thus, the time saved with this procedure could not fully compensate for the lower yield compared to the classical Dhara′s method [185], but MWs may be exploited for syntheses employing radioactive ^195m^Pt, thus requiring fast procedures.

Seven papers reported cycloaddition reactions involving Pt(II) complexes or intermediates. Coordinated organonitrile ligands allow for the direct synthesis of (imine)platinum(II) complexes by iminoacylation of ketoximes. These reactions are greatly accelerated by MW irradiation to give a mixture of *cis*- and *trans*-imino Pt(II) complexes, with high yields (ca. 75% in only 1–2 min vs. 47–62% in 15 min with CH) [186].

The coordinated CH_3_CH_2_CN in Pt(II) or Pt(IV) complexes undergoes [2 + 3] cycloadditions with cyclic nonaromatic nitrones, and these reactions were greatly accelerated (5 min–3 h under mild conditions) by focused MW irradiation to produce complexes with bicyclic oxadiazolines [187]. Similarly, the coupling of coordinated nitriles in *trans*-[PtCl_2_(NCCH_2_R)_2_] (R = CH_3_CO_2_ or Cl) complexes with nitrones, traditionally carried out in refluxing CH_2_Cl_2_ for 8 h to obtain the corresponding oxadiazoline Pt(II) complexes, was drastically accelerated with MW irradiation (1 h, 60 °C) while maintaining similar yields [188].

As reported for Pd complexes (Section 4.7), diazidoplatinum(II) complexes treated with organonitriles turned into bis(tetrazolato) complexes through 1,3-dipolar cycloaddition [189]. The reactions were performed under CH or with MW irradiation. Microwaves greatly accelerated the reactions (from 12 h to 1 h) while maintaining the yields, and, with propionitrile, the selectivity towards the expected product was increased. Similarly, bis(tetrazolato)platinum(II) complexes containing 1,3,5-triaza-7-phosphaadamantane (PTA) were obtained [190]. Likewise, the diazide platinum(II) complex, [Pt(N_3_)_2_(PPh_3_)_2_] reacted with 4-fluorobenzonitrile under MW irradiation to give *trans*-bis [5-(4-fluorophenyl)tetrazolato]bis(triphenylphosphane)platinum(II) [191].

The synthesis of Pt(II) complexes bearing one or two oxadiazolines was performed by cycloaddition of nitrones to coordinate nitriles in [PtCl_2_(PhCN)_2_] [192]. Under MW irradiation the first cycloaddition in the complex *trans*-[PtCl_2_(PhCN)_2_] was complete in 20 min (vs. overnight CH), with yields and selectivity similar to those obtained with CH. However, the two nitriles have different reactivity toward cycloaddition with nitrones under both thermal and MW conditions. Thus, the second cycloaddition with MWs was completed in 2.5 h. Microwave irradiation enhanced the reaction rates and rendered the reaction more selective because the first cycloaddition was accelerated to a greater extent than the second one.

The MAS between K_2_[PtCl_4_] and a series of bis(phosphanes) gave clean products with yields ≥65% in shorter reaction times compared to time-consuming and laborious traditional methods [193]. Similarly, the one-pot synthesis of *trans* mono- or diarylalkynyl substituted Pt(II) compounds containing phosphane or phosphite was developed with MWs simply starting from PtCl_2_ and ligands (with CuI in the case of bis-substitutions) without requiring the synthesis of intermediates [194].

Finally, MAS of Pd(II) and Pt(II) complexes with 3-acetyl-2,5-dimethylthiophene thiosemicarbazone and 3-acetyl-2,5- dimethylthiophene semicarbazone (**33**, Figure 11) resulted in lower reaction times and generally higher yield with respect to the CH method (see also Section 4.7) [176].

MAS was also used to prepare octahedral Pt(IV) complexes. Oxidation of cisplatin or ^15^N-cisplatin with hydrogen peroxide to give oxoplatin (**36**, Figure 11) was speeded up from the conventional 2 h [195,196] to a 5 min ramp period followed by 15 min at 70 °C under MWs (90% yield) [197,198].

**Figure 12:**
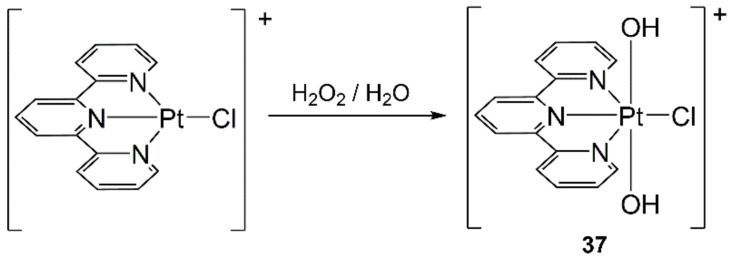
Scheme of the oxidation reaction of [PtCl(terpy)]^+^ (terpy = 2,2′:6′,2″-terpyridine).

Furthermore, the oxidation of [PtCl(terpy)]^+^ took advantage of MW heating. The oxidation of this complex was attempted with several oxidizing agents and under different experimental conditions to obtain a Pt(IV) complex suitable for drug targeting and delivery purposes. The best compromise in terms of yield and purity was obtained by a MW-assisted reaction at 70 °C in 50% aqueous H_2_O_2_ for 2 h to give compound **37** (Figure 12) in 82% yield. In that case, MW heating allowed a reaction that was unsuccessful with traditional heating [199].

Reaction of Pt(IV) complexes that contain one or two hydroxide ligands with acyl chlorides in acetone, in the presence of pyridine, was faster with MW heating (reflux overnight vs. heating the MW vessel to 55 °C over a 5 min ramp period and then holding at this temperature for 1 h at 50 W) [197,200,201,202,203,204].

MWs were also applied to investigate the kinetics of the reductive elimination of the organometallic compound [Pt(CH_3_)_3_(dppe)(O_2_CCH_3_)] compared to CH. Such a reaction was chosen as a probe of nonthermal effects in MAS by virtue of a polarized transition state and solvent with poor MW absorptivity (thus requiring high MW power). However, no evidence of nonthermal effects was observed [205].

### 4.9. Synthesis of a Pt(IV) Complex: An Unpublished (and Not Completely Satisfactory) Case Study

Within a wider project dealing with the synthesis of reactive Pt(IV) intermediates, we tried to apply MW to the oxidation of cisplatin with aqueous hydrogen peroxide (50% *w*/*w*) in EtOH to produce complex *cis*,*cis*,*trans*-[PtCl_2_(NH_3_)_2_(OH)(OCH_2_CH_3_)] (**38**, Figure 11). The compound contains an axial OH group that can be further esterified to give other derivatives (Figure 10).

The output of this synthesis is affected by the possible formation of byproducts as a result of the oxidation involving water as a source of one axial ligand or the reaction between EtOH and hydrogen peroxide.

In order to find the best conditions and, more importantly, to limit the number of experiments to be done, we chose to rationalize the syntheses applying a statistical design of the experiments (DoE). This is a methodology developed in 1958 by the British statistician Ronald Fisher consisting of an appropriate statistical analysis before performing the experiment to obtain as much information as possible from a minimum number of tests [206,207]. The DoE is widely used when a “chemical process” must be optimized, but surprisingly, it is rarely applied in inorganic labs, where the serendipitous trial-and-error approach is still used.

Among the possible DoE, we chose the factorial design. This is a set of experiments designed to allow researchers to study the effects that two or more “factors” (in our case, the experimental parameters) can have on a “response” (in our case, the yield).

Each factor has discrete possible values or “levels” and, usually, has assigned two levels (low and high). In a full factorial design, researchers measure responses at all possible combinations of levels for all factors. Such a DoE allows the investigator to study the effect of each factor, as well as the effects of the interaction between factors, on the final response [208].

For our DoE, we considered the following factors: (i) H_2_O_2_/Pt mole ratio, (ii) temperature, and (iii) reaction time. A three-factor, two-level (low and high) factorial design requires eight (i.e., 2^3^) experiments. This means carrying out the syntheses with the eight combinations of factors (see Appendix A for further details). At this stage, the Yates algorithm was applied to the experimental data, generating least squares estimates to identify the factors that have the most effect on the yield [209].

In our case, the analysis of the effects showed that an increase of reaction time and H_2_O_2_/Pt mole ratio increased the yield, whereas an increase in temperature had the opposite effect. The corresponding least squares model has coefficient of determination R^2^ = 0.92. Unfortunately, the 32% yield obtained with the best MW conditions in our DoE (H_2_O_2_/Pt mole ratio = 132; temperature = 60 °C, reaction time = 15 min) did not equal that of the CH (H_2_O_2_/Pt mole ratio = 132; temperature = 70 °C, reaction time = 5 h, yield = 80%) [210]. However, the statistical approach was not completely unsuccessful. In fact, the DoE indicates the influence of the factors on the yield and, more importantly, can provide the mathematical model that can predict the yield on the basis of different values of the factors. This represents a starting point for a focused design of new experiments.

### 4.10. Coinage Metals

Copper appears frequently in the series of first-row transition metal ions used to test the reactivity of a specific ligand or family of ligands, in particular with Schiff bases (see previous Section 3.2, Section 3.3 and Section 4.1 for details) [69,70,71,82,86,141,142,143,144,145,146,147,148,151].

Another paper reported the synthesis of sixteen Cu(II) complexes with Schiff bases derived from salicylaldehydes and L-amino acids by using an MW apparatus. The conventional solution method (in MeOH, 40 °C) took approximately 2 + 2 h to complete the two-step reaction scheme, whereas under MW irradiation, the complexes were obtained in 10 min by one-pot synthesis (in MeOH, 85 °C). The MW irradiation resulted to be effective (higher yields) for four complexes due to the presence of soluble leucine and electron-withdrawing dichlorosalicylaldehyde. For the other complexes, MAS yields were comparable to or even lower than those obtained by CH method [211].

The reaction between CuCl_2_ and 4-chloro- or 4-fluoro-1,2-phenylenediamine produced the monometallic complexes of the type “[CuL_2_]Cl_2_”. The following reaction of [CuL_2_]Cl_2_ with organotin dichlorides R_2_SnCl_2_ (R = C_6_H_5_, CH_3_) gave the four “[CuL_2_(SnR_2_)_2_]Cl_4_” complexes (reagent in stoichiometric amounts heated in MeOH by MW or CH). The usual reduction in the volume of solvent (from 30–60 mL to 3–5 mL) and reaction times (from 3–8 h to 5–8 min) accompanied by the increase in yield (63–70% to 83–90%) was observed by passing from CH to MW heating [212].

**Figure 13:**
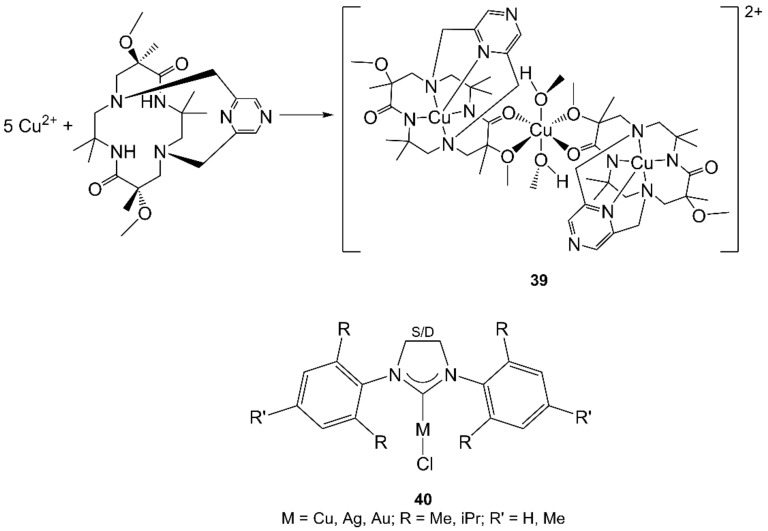
A selection of complexes containing coinage metals cited in the text.

One of the few examples of a different reaction pathway passing from CH to MAS is represented by the complexation of a pyrazine-capped 5,12-dioxocyclam to Cu(II) (cyclam = 1,4,8,11-tetraazacyclotetradecane). The reaction between the ligand and Cu(BF_4_)_2_ in refluxing MeOH containing K_2_CO_3_ (24 h) gave the expected metal complex with 1:1 stoichiometry (**39**, Figure 13). On the contrary, when the mixture was irradiated for 2 min in a consumer MW oven in the attempt to decrease the reaction time required for complexation, a trinuclear complex having an octahedral Cu(II) center complexed to two pyrazine−cyclam Cu units through the amide carbonyl oxygen and the methoxyl group oxygen of the cyclam unit was obtained. The latter complex was formed under MW conditions only [213].

Another multicopper complex was obtained by MW-assisted reaction between equimolar quantities of 4,4′-bipyridine (4,4′-bpy) and CuSO_4_ in water for 3 min in a household MW oven. The final complex is a one-dimensional polymer in which 4,4′-bpy acts as a bridging ligand supporting the formation of infinite [Cu(4,4′-bpy)(H_2_O)_3_(SO_4_)] chains packed in a 3D network via multi-hydrogen bonds [214].

A mixture of Cu(NO_3_)_2_ and bis(4-pyridylthio)methane (4-bpytm) (1:2 M:L ratio) was heated under reflux in EtOH for 21 h and [Cu(NO_3_)_2_(4-bpytm)_2_]·H_2_O was obtained in a yield of 98%. A mixture of Cu(NO_3_)_2_ and 4-bpytm (the same ratio) was irradiated for 90 s at 700 W in a DMF:EtOH (2:1) solution, and [Cu(NO_3_)_2_(4-bpytm)_2_]·solvent was obtained in 69% yield. The reaction of Cu(II) under the two different synthetic conditions afforded two 2D pseudo-polymorphs with different topology: the rhombic-grid in [Cu(NO_3_)_2_(4-bpytm)_2_]·H_2_O and the “parquet motif” in [Cu(NO_3_)_2_(4-bpytm)_2_]·solvent, being the non-rigid 4-bpytm spacer the controller of the dimensionality and topology of the resulting coordination polymer, but the mixture of solvents used seemed to play a template role [215].

Bimodal agents incorporating two metal ions were designed to have a paramagnetic metal complex (Mn(II) and Gd(III)) for magnetic resonance imaging (MRI) “clicked” to a second moiety containing a radiometal complex (cold Cu(II), Ga(III), In(III)) for nuclear medicine applications. The two molecular entities to be linked were, on the one hand, a propargyl-DOTA-tris(*t*-Bu) ester and a propargyl-NOTA(*t*-Bu)_2_ (DOTA = 1,4,7,10-tetraazacyclododecane-1,4,7,10-tetraacetic acid; NOTA = 1,4,7-triazacyclononane-1,4,7-triacetic acid) and, on the other hand, two azidocorroles. The azidocorroles were complexed with Cu(II), Ga(III), In(III), and Mn(II), whereas Gd(II), Ga(III), and Cu(II) were used for the tetraazamacrocycles before the click reaction. The click reactions were carried out in DMF, using excess of the alkyne derivative in the presence of the azido counterpart, CuI and *N*,*N*-diisopropylethylamine (DIPEA). Surprisingly, a very slow progress of the reaction was observed and the attempt to increase the temperature of the reaction mixture to 50 °C resulted in the degradation of corrole over time. On the contrary, when the same mixture was irradiated in a sealed quartz vessel using an MW oven at 60 W (50 °C) for 30 min, the final complexes were obtained (with variable yield, from 26% to 80%) [216].

In the continuous search for a drastic reduction in the reaction times and energy employed, MAS was applied to the synthesis of twelve NHCs of group 11 metals [MCl(NHC)] (M = Cu, Ag, Au, **40**, Figure 13). The CH produced the complexes with very good yields (>70%) at refluxing temperatures (in toluene or water) after 24 h. Attempts to speed up the synthesis in water by applying MW heating reduced the reaction times to about 4–5 h in the best cases. The change of solvent to THF allowed to synthesize the complexes in comparable yields but in 30 min only (at 110 °C) [217].

Six examples of Au(I) complexes of general formula [AuCl(N–N)][PF_6_] and three examples of organometallic [AuCl(C–N)] (N-N = bpy-type ligands; C–N = cyclometalated 2-phenylpyridine-type ligands) were successfully prepared by reacting HAuCl_4_, NaPF_6_, and the ligands (1:3:1 mole ratio; solvent: acetonitrile/water 1:5 or water alone) in sealed vessels under MW heating. The reaction to obtain the coordination compounds was carried out at 110–120 °C for 10–30 min, whereas the cyclometalated Au complexes needed slightly harsher conditions (140–160 °C for 20–60 min), but in any case, they represented a substantial improvement over conventional procedures [218].

Finally, another example of the application of MAS to Au complexes was reported in the Section 3.2 [62].

### 4.11. Zinc and Mercury

As in the case of copper, Zn(II) (and less frequently Hg(II)) is also a common metal ion that was often used with other transition metal ions to produce coordination compounds (see previous Section 3.2, Section 3.3, Section 4.1 and Section 4.4 for details) [69,82,86,87,147,148,151]

### 4.12. Lanthanides

Although lanthanides are not strictly considered “transition metals”, they were added to the present discussion because f-block metal complexes are interesting for their magnetic and luminescent properties for medical diagnostics, luminescent imaging, and biochemistry, and some examples of MAS were reported.

Using MW heating, [Ln(TTA)_3_(TPPO)_2_] (Ln = La(III), Eu(III), Tb(III) and Tm(III), TTA = 2-thenoyltrifluoroacetone, and TPPO = triphenylphosphane oxide) complexes of interest for luminescence applications were synthesized in few minutes with minimal purification steps and yields (40–80%) comparable to literature values. In particular, a mixture of TTA, TPPO, and Ln(III) in water-isopropanol (in 3:2:1 molar ratio) was heated in an MW reactor to 100 °C for 1–20 min [219].

MW heating was also used to modify the coordinated ligand through CuAAC reactions in Ln(III) complexes (Ln = La, Eu, and Tb) for luminescence applications. MW irradiation accelerated the reactions, reducing reaction times (15–60 min at 100 °C) with yields from moderate to very good for the isolated products. This procedure also allowed the reaction of alkynyl cyclen triamides complexes that previously failed to react despite forcing (traditional) conditions. The synthesis of clicked heteromultimetallic complexes was also carried out (30 min, 100 °C) combining different complexes with alkyne or azide reactive groups [220]. Another example of clicked Gd(III) complexes was reported in Section 4.9 [216].

With the aim of preparing complexes with antifungal activity, [(2-hydroxybenzaldehyde)-3-isatin]bishydrazone (HISA) was synthesized (via MAS) and used in the reaction with Ln(III) chloride (Ln = La, Ce, Pr, Nd, Sm, Eu, or Gd) to give [Ln(HISA)_2_Cl_3_] [221]. Ligand and LnCl_3_ were mixed and dissolved in MeOH at pH 6.5; after evaporating the solvent, the mixture was heated for 10 min (instead of refluxing MeOH 10–12 h) in the MW oven, resulting in a yield of 60–70%.

Finally, 2-phosphonoethanesulfonic acid was used to prepare Ln (Ln = Ho, Er, Tm, Yb, Lu, Y) complexes by MW-assisted heating (170 °C, 2 h). The yields varied from 25 to 56% [222]. It was observed that stirring during the reaction led to lower yields, whereas increasing the reaction time at constant stirring rate led to higher yields.

## 5. Conclusions

What results from the analysis of over 150 papers carried out here is that, for years, MW irradiation has been proving its value as a useful synthetic tool within the coordination and organometallic chemistry community but probably with results that are less captivating with respect to other fields.

It is evident that the main outcome, common to all the mentioned experiments, is that MW pushes reactions to completion more rapidly than CH. In all examples, there is a change in the timescale, roughly speaking, from hours to minutes.

The efficient transfer of energy into the reaction medium contributes to the rapid heating, resulting in a uniformly reached temperature in seconds. On the contrary, in CH, the heating plate must heat the glassware, the oil, or sand bath possibly present, and, only then, the reaction mixture with gradient of temperature throughout the space. Furthermore, the pioneers of MW in inorganic chemistry Mike Mingos and David Baghurst discovered, already in 1992, the concept of superheating, a phenomenon whereby MWs heat up solvents above their normal boiling points, which further contributes to kinetics.

In many of the references, one of the reagents is the solvent itself. This experimental condition together with the fact that the solvent is generally present in a lower quantity with respect to CH (so that reactant concentrations are higher under MW heating) speeds up the reaction rate.

We have observed that simple domestic MW ovens are still used not only in the least recent papers. In such ovens, where large cavities require unspecific glassware, which are heated more or less like a cup of coffee, the control of the experimental setting is limited (e.g., no stirring, weak control of temperature except under reflux). Evidently, in these conditions, the MW ovens are simply used as sophisticated hotplates. The evolution to the single-mode microwave systems allowed to decrease, focus, and regulate the power used to reach and maintain a certain temperature value.

Moreover, controlled MW heating under sealed vessel conditions, sometimes with in situ systems to control the internal pressure as well as the temperature (also by using an external cooling flow of air), make possible to raise the bar. In the closed vessel, the pressure is significantly enhanced, and the reaction temperature is not limited by the atmospheric boiling point. In fact, as we just saw, the reactions that take a real advantage from MW use are those that require harsh thermal condition. The possibility of optimizing and increasing energy shortens reaction times and improves reproducibility without or with limited formation of side products. Clearly, several hours at very high temperature by CH increase the risk of decomposition of reagents or products and the development of unwanted side reactions, decreasing yields and purities and increasing the amount of by-products. On the other hand, only in a few cases was the use of MAS disappointing, with the formation of mixtures of products or with lower yields than with CH.

What is less stimulating from the papers examined in this review article is that the experimental data do not allow to say that MW has a specific effect on the reaction. In other words, the number of times where MAS produced compounds different from CH can be counted on the fingers of two hands (roughly, in less than 10% of the references). The more frequent selectivity or specificity is related to the (repeatedly mentioned) possibility of performing synthesis requiring such demanding conditions that are extremely difficult (if not impossible) to reach simply by CH without extensive decomposition.

In other words, from the limited analysis of the present review, MW heating can improve the rate of reactions because a more efficient heating can enhance the rate of reactions, whereas the MW irradiation is probably unable to “promote” particular reactions by nonthermal effects.

All these factors should not make us to forget that, for years, MWs unequivocally have been proving their utility as both a time-saving tool and a novel means of performing challenging transformations. Microwave systems will continue to evolve to meet the changing needs of synthetic chemists as well as of scientists in other areas, and it is hoped that new doors will be opened to perform novel transformations also in the field of coordination and organometallic chemistry. Thus, when planning a new inorganic synthesis, it is worth trying with MW irradiation.

## Figures and Tables

**Figure A1:**
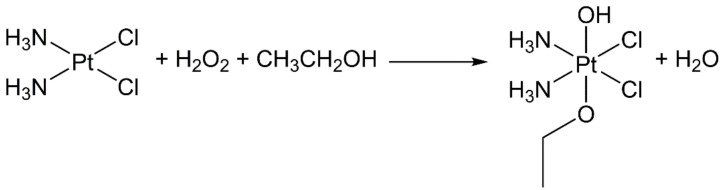
Reaction scheme of the synthesis of (*OC*-6-44)-diamminedichloridoethanolatohydroxidoplatinum(IV).

**Figure A2:**
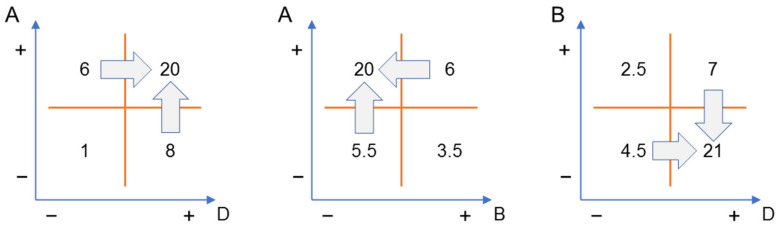
The matrices show the mean values of the yields obtained at the different levels of the factors, considered two by two. The “−“ and “+” symbols represent the lowest and the highest values of the factors (A = H_2_O_2_/Pt mole ratio, B = temperature, D = reaction time), respectively. The arrows point towards the higher yields, indicating the best level for the factors.

## Appendix A

### Experimental Section

The chemicals used for the synthesis of *cis*,*cis*,*trans*-[PtCl_2_(NH_3_)_2_(OH)(OCH_2_CH_3_)] (See Section 4.9), or (*OC*-6-44)-diamminedichloridoethanolatohydroxidoplatinum(IV) according to the IUPAC nomenclature, were obtained from common commercial sources and were used as received and without further purification. Cisplatin was synthesized with the Dhara′s method [185]. Microwave irradiation was performed by using a CEM Discover^®^ SP System equipped with a focused single-mode and self-tuning cavity, an air-cooling system, and an automated power control based on temperature feedback, providing power in increments of 1 W from 0 to 300 W.

In a typical experiment, 12 mg of cisplatin and the chosen amount of H_2_O_2_ 50% *w*/*w* were introduced into a 10 mL MW glass vessel with 3 mL of ethanol (Figure A1). After capping it, the vessel was introduced into the MW oven, heated to the selected temperature over a 5 min ramp period, and then kept at this temperature for the desired time under stirring (power 20 W).

For the experimental design, the temperature levels were 60 and 68 °C, the H_2_O_2_/Pt mole ratio levels were 33 and 132, and the hold time levels were 5 and 15 min. After the reaction mixture was cooled to room temperature, the vessel was removed from the cavity, and the unreacted solid was removed by centrifugation. The solution was evaporated under vacuum with a rotary evaporator. After trituration of the residue with diethyl ether, the mixture was kept in the refrigerator overnight, and the resulting powdery product was isolated by centrifugation and dried under nitrogen flow.

The solid was analyzed by using a C18 Phenomenex Phenosphere-NEXT (5 μm, 250 × 4.6 mm ID) column on a Waters HPLC-MS instrument (equipped with Alliance 2695 separations module, 2487 dual lambda absorbance detector, and 3100 mass detector). The UV-visible detector was set at 210 nm. The eluent was a 70/30 mixture of 15 mM aqueous HCOOH/methanol, and the flow was 0.5 mL min^−1^. The amount of the Pt(IV) complex was quantified with a calibration curve.

The eight experiments produced eight sets of four values (i.e., H_2_O_2_/Pt mole ratio, temperature, reaction time, and yield) that, after range scaling, were analyzed by applying the Yates algorithm using a Microsoft Excel spreadsheet [223]. The final mathematical model (R^2^ = 0.92) is reported in Equation (A1):

Yield = 8.75 + 4.25 × A − 4 × B − 3 × C + 5.25 × D − 3 × E (A1)

where A = H_2_O_2_/Pt mole ratio, B = temperature, C = H_2_O_2_/Pt mole ratio × temperature, D = reaction time, E = temperature × reaction time. A positive sign indicates a positive effect on the response, whereas a negative sign is detrimental.

For a more qualitative and intuitive analysis of the effect of factors, the following plots were also produced (Figure A2). The matrices show the mean values of the responses (yields) obtained at the different levels of the factors, considered two by two.

In the plots of Figure A2, the analysis of the effects shows that an increase of reaction time and H_2_O_2_/Pt mole ratio enhances the yield, whereas an increase in temperature has the opposite effect. This is in tune with what was obtained in Equation (A1) that, in addition, contains the effect of combined factors C and E.
